# Investigating the optoelectronic properties and photovoltaic performance of Na_2_AuGaBr_6_ based double perovskite solar cells via numerical simulation and AI techniques

**DOI:** 10.1038/s41598-026-41519-x

**Published:** 2026-02-27

**Authors:** Bipul Chandra Biswas, Asadul Islam Shimul, Indrojit Paul, S. AlFaify, Mohamed Benghanem, Md. Azizur Rahman, Gideon F. B. Solre, Noureddine Elboughdiri

**Affiliations:** 1 Department of Electrical and Electronic Engineering, Gopalganj Science and Technology University, Gopalganj, 8105 Bangladesh; 2 Department of Physics, College of Sciences, King Khalid University, P.O. Box 960, AlQura’a, Abha, Saudi Arabia; 3 Physics Department, Faculty of Science, Islamic University of Madinah, Madinah, 42351 Saudi Arabia; 4 Department of Electrical and Electronic Engineering, Begum Rokeya University, Rangpur, 5400 Bangladesh; 5 Department of Chemistry, Thomas J. R. Faulkner College of Science, Technology, Environment and Climate Change, University of Liberia, Monrovia, 00231 Montserrado County Liberia; 6 Chemical Engineering Department, College of Engineering, University of Ha’il, P.O. Box 2440, Ha’il, 81441 Saudi Arabia

**Keywords:** Na_2_AuGaBr_6_, Double perovskite solar cell, Machine learning, Deep learning, SCAPS-1D, Energy science and technology, Materials science, Physics

## Abstract

This study offers a thorough analysis of Density Functional Theory (DFT) and SCAPS-1D to assess the optoelectronic performance of cubic Na_2_AuGaBr_6_ double perovskites, highlighting its potential for advanced optoelectronic and photovoltaic applications. Device simulations were methodically conducted utilizing Na_2_AuGaBr_6_ as the active absorber material, in conjunction with various electron transport layers (ETLs) including TiO_2_, ZnO, WS_2_, C_60_, IGZO, and In_2_S_3_, as well as hole transport layers (HTLs) such as CuI, CFTS, NiO, CuSbS_2_, V_2_O_5_, Sb_2_S_3_, MoTe_2_, and CuO, to ascertain the optimal device configuration. Comprehensive parametric optimization was performed by analyzing the effects of different left (Co, Ni, Au, Pt, Pd, and Se) and right (Ca, Ba, Mg, Ag, Al, and Cr) metal contacts, band alignment, layer thicknesses, interface and bulk defect concentrations, as well as temperature on the overall photovoltaic performance. Of the 48 simulated device structures, the Al/FTO/WS_2_/Na_2_AuGaBr_6_/V_2_O_5_/Ni configuration demonstrated superior performance, achieving a power conversion efficiency (PCE) of 28.96%. Additionally, advanced machine learning (ML) and deep learning (DL) models were utilized to forecast and corroborate device performance trends. Of the eleven methods evaluated, the Gradient Boosting model exhibited remarkable predictive accuracy, attaining a R^2^ of 0.954 and a negligible mean absolute percentage error (MAPE) of 0.0218. These findings affirm the significant promise of Na_2_AuGaBr_6_-based perovskites for lead-free, high-efficiency solar systems and establish ML and DL-assisted modeling as an efficient method for performance improvement and material design in photovoltaic research.

**Supplementary Information:**

The online version contains supplementary material available at 10.1038/s41598-026-41519-x.

## Introduction

Photovoltaic (PV) technologies provide a very promising and environmentally viable method for renewable energy generation. Increasing investment in renewable energy systems is crucial for mitigating climate change and promoting long-term global sustainability^1,2^. To augment the acceptance and competitiveness of solar technologies, it is essential to reduce power generation costs while lowering greenhouse gas emissions. In this situation, perovskite solar cells (PSCs) have rapidly emerged as a highly efficient and economical photovoltaic technology, attaining performance levels akin to traditional silicon-based solar cells due to substantial advancements in material engineering, fabrication techniques, and device architecture^3^. Recently, lead-free double perovskites (A_2_BB′X_6_), with A representing rare-earth or alkali cations, B and B′ denoting transition metal cations, and X signifying halide anions, have attracted significant interest as next-generation photovoltaic materials due to their advantageous optoelectronic characteristics and diminished environmental toxicity^4,5^. These compounds demonstrate elevated absorption coefficients, adjustable bandgaps, minimal exciton binding energies, superior charge-carrier mobility, extended carrier lifetimes, and robust defect tolerance^6^. The efficiency of double perovskite solar cells (DPSCs) have significantly increased from approximately 3.5% to almost 27%, highlighting their potential to compete with or supplant traditional silicon-based solar cells^7,8^. In contrast to silicon solar cells, which necessitate intricate and expensive production methods, perovskite materials can be produced using economical solution-processing techniques, facilitating scalable and adaptable device fabrication. Nonetheless, lead-based perovskites pose environmental and health risks owing to lead toxicity and solubility. As a result, lead-free double perovskites have developed as sustainable alternatives, substituting hazardous Pb^2+^ ions with harmless cations, thereby providing enhanced structural flexibility, chemical stability, and environmentally acceptable avenues for high-performance solar applications^9,10^.

Recent studies have underscored the increasing importance of gold (Au) and gallium (Ga)-based double perovskite halides as potential materials for optoelectronic and thermoelectric applications. Ayyaz et al.^11^ indicated that Na_2_AuMX_6_ (M = Al, Ga; X = Br, I) compounds possess stable cubic structures, mechanical durability, and direct band gaps (0.22–1.82 eV), with bromide variants demonstrating significant visible absorption appropriate for solar cells and iodide counterparts enhancing infrared optoelectronics. Albalawi^12^ examined Cs_2_AuXZ_6_ (X = Al/Ga; Z = Cl/Br) perovskites, affirming their ductility, thermal stability, and advantageous band gaps (0.84–1.86 eV) for solar applications, whereas Cs_2_AuGaBr_6_ exhibited metallic conductivity suitable for charge transport. Usman et al.^13^ investigated A_2_AuSbZ_6_ (A = Na, K; Z = F, Cl), uncovering optimum band gaps (0.67–1.30 eV), significant photon absorption, and robust thermoelectric performance, rendering them appropriate for integrated solar and energy-harvesting technologies. Ayub et al.^14^ investigated Na_2_XGaF_6_ (X = In, Tl) perovskites, revealing substantial band gaps (2.6–5.84 eV) and enhanced thermoelectric performance with increasing temperature, especially for Na_2_TlGaF_6_ under elevated thermal settings. Moreover, Assiouan et al.^15^ established the stability and favorable optoelectronic characteristics of lead-free Rb_2_AuBiX_6_ (X = Br, Cl, F), exhibiting band gaps ranging from 0.17 to 1.08 eV and significant ZT values (0.33–0.66). These investigations collectively highlight that Au and Ga-based double perovskite halides exhibit structural stability, robust optical absorption, and effective thermoelectric performance, designating them as multifunctional materials for advanced optoelectronic and renewable energy applications.

Recent advancements in computational modeling have increasingly highlighted the utilization of SCAPS-1D simulations to forecast and enhance the PV performance of innovative lead-free and environmentally sustainable PSCs. Elyazid^16^ performed a numerical evaluation of a Na_2_AuGaBr_6_/CuSbS_2_ double-absorber PSC, with an impressive PCE of 28.33%. The incorporation of a Beta MPPT controller enhanced power extraction and operational stability, confirming its promise for high-efficiency, environmentally sustainable solar energy systems. Hakami^17^ investigated the FTO/TiO_2_/Cs_2_AgBiBr_6_/NiO/Au configuration with SCAPS-1D, revealing that the lead-free Cs_2_AgBiBr_6_ absorber achieved a PCE of 26.71%, thereby illustrating the efficacy of interface and material optimization in improving device performance. Raj et al.^18,19^ presented advanced lead-free PSC structures to improve efficiency and stability. A bilayer absorber system combining Cs_2_SnBr_6_ and Cs_2_TeI_6_ attained an efficiency of 38.62% via complementary broad-spectrum absorption, while machine learning identified performance fluctuations produced by humidity. A solar cell based on Cs_2_AgBiBr_6_, utilizing TiO_2_/PCBM/SnO_2_ and Cu_2_O/NiO/Spiro-MeOTAD transport layers, exhibited impressive PV performance (PCE = 27.78%) and stable operation under varying temperature, humidity, and interior illumination conditions, facilitating IoT applications. Benhafsa et al.^20^ examined Ba_2_AgBiX_6_ (X = I, Br, Cl) perovskites, establishing their thermal stability and indirect semiconductor properties, with Ba_2_AgBiBr_6_ achieving a PCE of 13.93%. Ayyaz et al.^21^ indicated that A_2_NaAlI_6_ (A = Rb, Cs) demonstrates straight band gaps (~ 1.7–1.78 eV) and attained efficiencies of up to 28% in ITO/CSTO/A_2_NaAlI_6_/CuAlO_2_ combinations. Thakur et al.^22^ investigated K_2_XAuCl_6_ (X = Al, Ga), revealing that K_2_AlAuCl_6_-based tandem cells attained a J_SC_ of 12.85 mA/cm^2^, PCE of 22.39%, and V_OC_ of 2.20 V. Deswal et al.^23^ showed remarkable defect tolerance in Cs_2_AgInBr_6_, achieving PCEs of 29.49% at 200 K and 26.9% at 300 K, whilst Khan et al.^24^ forecasted values of 30–32% for X_2_MgGeI_6_ (X = Rb, Cs) owing to robust optical absorption and favorable band gaps, highlighting its potential for next-generation inorganic PSCs.

This study provides a comprehensive investigation of the optoelectronic characteristics of Na_2_AuGaBr_6_ halide perovskites through DFT and SCAPS-1D simulations to evaluate their potential for photovoltaic applications. The Na_2_AuGaBr_6_-based DPSC was analyzed both with and without a HTL, demonstrating superior power conversion efficiency and structural integrity. The optimized device configuration, Al/FTO/WS_2_/Na_2_AuGaBr_6_/V_2_O_5_/Ni, demonstrated elevated photo response and enhanced charge transport characteristics, validating its viability as a lead-free, eco-friendly substitute for traditional PSCs. Parametric adjustment of absorber thickness, doping concentrations, defect densities, and temperature revealed that reducing interfacial defects substantially improves carrier recombination kinetics and overall device efficiency. Simulated current density-voltage (J-V) and quantum efficiency (QE) profiles offered significant insights into light absorption and charge extraction methods. Furthermore, three ML models and eight DL models were utilized to forecast device performance, with Gradient Boosting (GB) attaining the highest accuracy and recognizing defect density as the principal determinant affecting efficiency. The amalgamation of DFT-derived material insights, SCAPS-1D device simulations, and ML-based predictive modeling creates a solid framework for enhancing Na_2_AuGaBr_6_ perovskites for scalable, lead-free, and high-efficiency solar energy applications.

## Computational methodology

The optoelectronic properties of Na_2_AuGaBr_6_ were systematically examined using DFT within the Cambridge Serial Total Energy Package (CASTEP)^25^. The Perdew–Burke–Ernzerhof (PBE) exchange–correlation functional, utilizing the generalized gradient approximation (GGA), was utilized to characterize electron–electron interactions^26^. PBE-GGA consistently underestimates absolute semiconductor band gaps; nonetheless, it accurately reflects relative electronic trends, band dispersion, and qualitative characteristics of the electronic structure. Due to its numerical stability, transferability, and computational efficiency, PBE is ideally suited for large supercells, defect-containing, and doped systems. Although newer methodologies like hybrid functionals and GW techniques enhance band gap precision, their significantly greater computational expense and potential overestimation of electrical properties restrict their use in large-scale simulations^27,28^. Consequently, PBE provides an optimal equilibrium between computing efficiency and physical precision. The electron-ion interactions were characterized using Vanderbilt-type ultrasoft pseudopotentials, providing an accurate and computationally efficient model of the system. A plane-wave cutoff energy of 400 eV was chosen to efficiently differentiate between valence and core electron contributions. A 10 × 10 × 10 Monkhorst-Pack k-point mesh was employed for Brillouin zone sampling to provide accurate convergence of the electronic structure calculations. The high k-point density enhances the precision of Fermi-level determination and facilitates the accurate resolution of intricate features near band extrema, which is especially crucial for materials displaying significant band dispersion or nuanced electronic transitions. The crystal shape was structurally optimized via the Broyden-Fletcher-Goldfarb-Shanno (BFGS) minimization technique, which iteratively reduced lattice constants and atomic coordinates until achieving the ground-state arrangement. The DFT calculations were converged with criteria of 1 × 10^− 5^ eV/atom energy, 0.05 GPa stress, 0.03 eV/Å force, and 0.001 Å displacement. The elastic constants of the system were determined using the stress-strain method^29^, facilitating a comprehensive knowledge of the mechanical stability and anisotropy of Na_2_AuGaBr_6_. To thoroughly evaluate the optoelectronic properties of the Na_2_AuGaBr_6_-based perovskite absorber, extensive simulations were conducted utilizing SCAPS-1D, which resolves Poisson’s and carrier continuity equations to examine charge transport, recombination, and potential distribution under steady-state conditions^30,31^. These simulations offered essential insights into charge carrier dynamics, band alignment, and internal electric field behavior, forming the basis for assessing overall device efficiency. Advanced ML and DL models were built to predict essential photovoltaic metrics, including PCE, FF, V_OC_, and J_SC_, thereby enhancing predictive understanding. Of the eleven methods evaluated, the Gradient Boosting (GB) model exhibited exceptional predictive accuracy and significant concordance with SCAPS-1D results, adeptly capturing nonlinear interactions among structural and material characteristics. The low error rates and strong generalization of the GB model confirm its dependability for predicting solar performance. The results highlight the significant potential of Na_2_AuGaBr_6_ as a lead-free, high-efficiency perovskite absorber and demonstrate the collaboration between numerical simulation and AI-driven modelling in enhancing sustainable solar research and design.

## Result and discussion

### DFT analysis

#### Structural properties

**Fig. 1:**
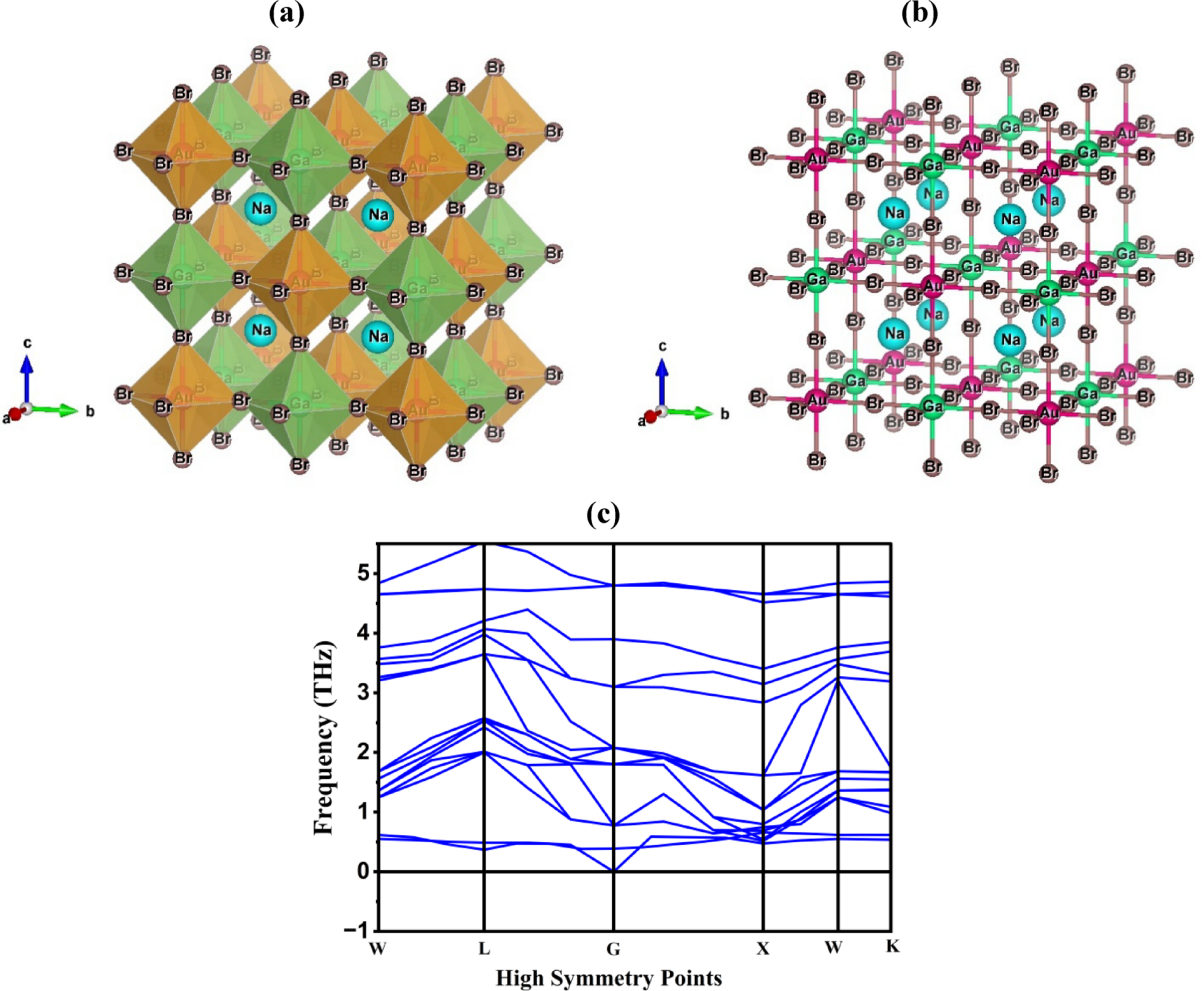
Structural and dynamical characterization of Na_2_AuGaBr_6_ showing (**a**) polyhedral geometry, (**b**) ball-and-stick crystal structure, and (**c**) phonon dispersion relations along high-symmetry directions.

The crystallographic investigation indicates that Na_2_AuGaBr_6_ crystallizes in a cubic perovskite structure inside the Fm-3 m (No. 225) space group, as depicted in Fig. 1a,b. The atomic arrangement within the unit cell is precisely delineated: Na atoms hold the Wyckoff position at fractional coordinates (0.75, 0.25, 0.25), Au atoms are situated at (0, 0, 0), Ga atoms are positioned at (0.5, 0, 0), and Br atoms are found at (0.75, 0, 0). This arrangement establishes a periodic lattice structure, essential for ascertaining the material’s electronic band dispersion, thereby yielding significant insights into its conductivity and potential optoelectronic properties. Following structural optimization, Na_2_AuGaBr_6_ demonstrated a refined lattice constant of 11.44 Å, closely corresponding with previously documented values for cubic double perovskites^11,32^. This agreement proves the computational procedure’s accuracy and establishes the material’s structural integrity. The Goldschmidt tolerance factor (t) was calculated using the known relation (Eq. 1) to assess the possibility of perovskite phase development^33^.

1 $$\:t=\:\frac{{r}_{A}+\:{r}_{X}}{\sqrt{2}\:(\frac{{r}_{B}+\:{r}_{B{\prime\:}}}{2}+\:{r}_{X})}$$

In this expression, r_A_, r_B_, r_B’_, and r_X_ denote the ionic radii of the Na, Au, Ga, and Br atom, respectively. Perovskite structures have optimal stability when t ranges from 0.8 to 1.0, with increased resilience noted as the value approaches one. The determined tolerance factor for Na_2_AuGaBr_6_ is t = 0.83, with ionic radii values r_A_ = 1.62, r_B_ = 1.51, r_B’_ = 0.76, and r_X_ = 1.82. The value of 0.83 is comfortably situated within the stability range documented in the literature (0.8 < t < 1.2)^34^, thereby affirming the structural integrity of the cubic phase. Furthermore, the thermodynamic stability of the molecule was evaluated through the formation energy (E_f_) using Eq. (2)^35^.

2 $$E_{f} = {\text{ }}E_{{Na2AuGaBr6}} - {\text{ }}\left( {2E_{{Na}} + {\text{ }}E_{{Au}} + {\text{ }}E_{{Ga}} + {\text{ }}6E_{{Br}} } \right)$$

Here, E_Na2AuGaBr6_ represents the total energy of the optimum configuration, whereas E_Na_, E_Au_, E_Ga_, and E_Br_ correspond to the energy of the individual constituent atoms. The formation energy of Na_2_AuGaBr_6_ was calculated to be -0.0025 eV/atom, indicating that the compound is energetically advantageous and thermodynamically stable. To examine dynamic stability, phonon dispersion calculations were performed after full optimization of atomic positions and lattice volume. The phonon dispersion relations illustrated in Fig. 1c were derived employing the finite displacement approach within the context of density functional perturbation theory, assessed along the high-symmetry path. The phonon spectrum exhibits smooth and distinct branching throughout the entire Brillouin zone, signifying stable vibrational modes and coherent lattice dynamics. The absence of imaginary (negative) phonon frequencies confirms the absence of soft modes or structural instabilities, thereby affirming the dynamical stability of Na_2_AuGaBr_6_ under ambient conditions and enhancing its suitability for optoelectronic and photovoltaic device applications^36^. Overall, the combination of an optimized lattice parameter, favorable tolerance factor, negative formation energy, and lack of soft phonon modes collectively affirm the structural, thermodynamic, and dynamic stability of Na_2_AuGaBr_6_. These attributes emphasize its feasibility for experimental synthesis and affirm its appropriateness for incorporation into solar, optoelectronic, and other sophisticated energy-related applications necessitating enduring structural integrity.

#### Electronic properties

**Fig. 2:**
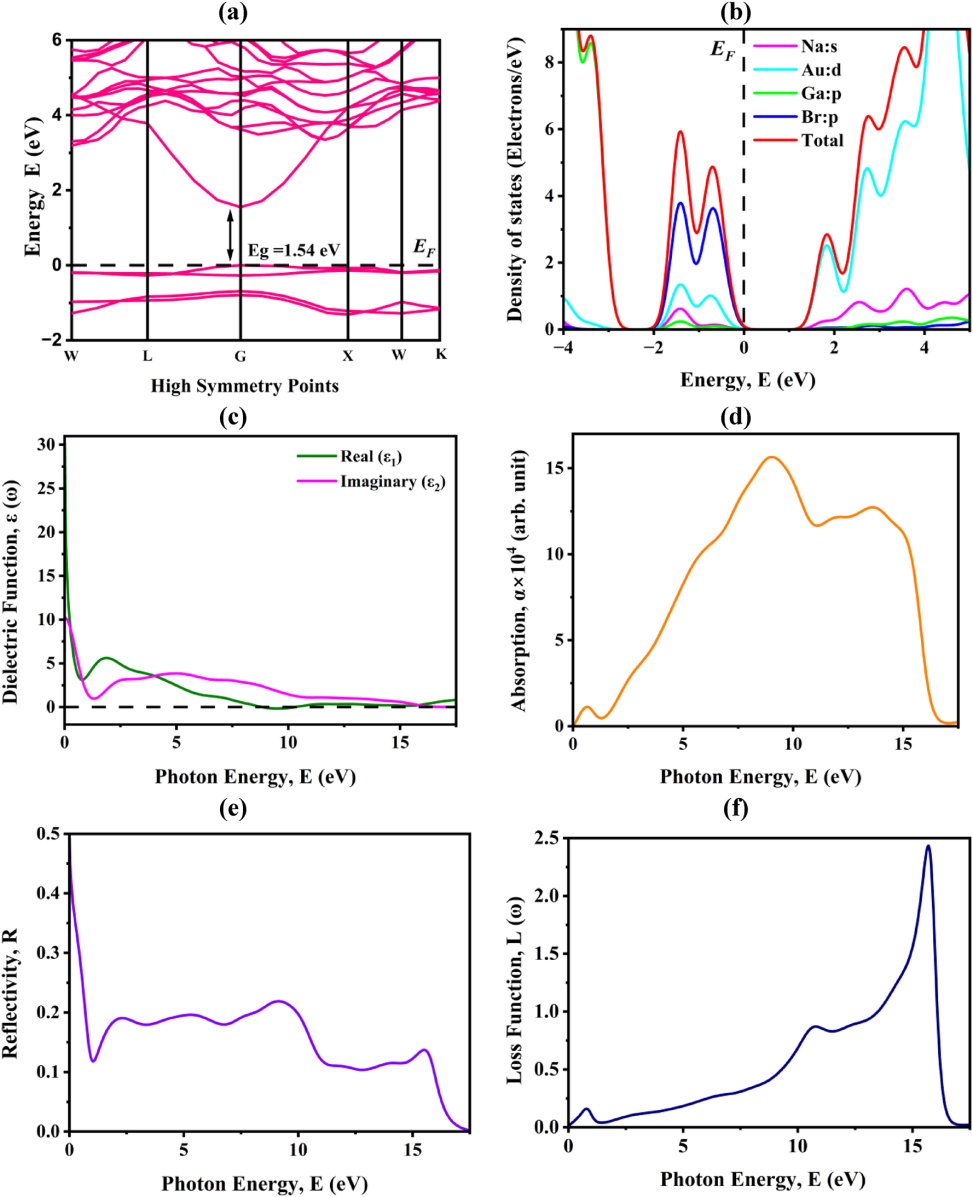
Computed electronic and optical features of Na_2_AuGaBr_6_ represented by (**a**) band structure, (**b**) PDOS, (**c**) dielectric function, (**d**) absorption, (**e**) reflectivity, and (**f**) energy loss function.

The examination of electronic properties offers essential understanding of charge dynamics and electronic interactions in crystalline solids, which directly influence their performance in optoelectronic and photovoltaic applications. Two essential descriptors in this context are the band structure and the density of states (DOS), both of which elucidate the intrinsic mechanisms regulating electron excitation, carrier transport, and overall conductivity. The electronic band structure of Na_2_AuGaBr_6_ was computed following complete structural optimization. Figure 2a illustrates that the dispersion curves exhibit significant high-symmetry regions across the Brillouin zone, which are essential for comprehending the electronic transport features. The electronic structure investigation confirmed that Na_2_AuGaBr_6_ is a direct bandgap semiconductor, with the valence band maximum (VBM) and conduction band minimum (CBM) situated at the Γ point, rendering it highly appropriates for optoelectronic and photovoltaic applications owing to its effective photon absorption and emission properties^37^. The bandgap, computed using PBE-GGA functional, was found to be 1.54 eV, aligning with previously reported values and affirming the robustness of the computational method^11,38^. The results indicate that Na_2_AuGaBr_6_ possesses significant potential as a lead-free, stable, and efficient absorber material for advanced optoelectronic and photovoltaic devices.

The partial density of states (PDOS) was assessed, offering a more nuanced understanding of the atomic contributions to electronic states. Figure 2b demonstrates that the DOS profile across the energy range of -4 eV to + 5 eV emphasizes the hybridization of Na, Au, Ga, and Br orbitals, excluding the bandgap area. At the valence band edge, the Ga-p orbitals predominate the electronic states, whilst the Br-p orbitals contribute insignificantly. In contrast, the conduction band area is predominantly influenced by Au-d orbitals, indicating a significant role of Au in determining optical and electronic transitions. This orbital hybridization highlights the intricate interaction of atomic states in determining the compound’s electrical properties. The comprehensive study reveals that Na_2_AuGaBr_6_ possesses a favorable direct bandgap and a clearly defined orbital contribution to its density of states, both of which are critical for effective charge separation and transport. The findings identify Na_2_AuGaBr_6_ as a viable double perovskite option for photovoltaic, optoelectronic, and light-harvesting applications, where stability and direct bandgap semiconductors are crucial for optimizing device efficiency.

#### Optical properties

An extensive examination of the optical response of Na_2_AuGaBr_6_ was conducted to assess its suitability for incorporation into optoelectronic and solar energy conversion devices. The optical parameters, such as the dielectric function, absorption coefficient, reflectivity, and electron energy-loss spectrum, provide essential insights into the material’s response to electromagnetic fields across a wide range of photon energies. The complex dielectric function, ε(ω), which governs the material’s interaction with incident light, is expressed in Eq. (3).

3 $$\varepsilon \left( \omega \right){\text{ }} = {\text{ }}\varepsilon _{1} \left( \omega \right){\text{ }} + {\text{ }}i\varepsilon _{2} \left( \omega \right)$$

where ε_1_(ω) signifies the real component, related to light dispersion, and ε_2_(ω) denotes the imaginary component, pertaining to absorption phenomena. The imaginary component, ε_2_(ω), was calculated utilizing momentum matrix elements, whereas ε_1_(ω) was determined via the Kramers–Kronig relation^39,40^. Figure 2c depicts the dielectric spectra of cubic Na_2_AuGaBr_6_ up to a photon energy of 30 eV. The static dielectric constant, ε1(0), is determined to be 30.17, signifying a substantial ability for polarization in response to an applied electric field. Simultaneously, ε_2_(ω) exhibits a pronounced decline as photon energy increases, with significant contributions extending to 15.5 eV, indicating the material’s robust absorption capacity in the low-energy spectrum.

The absorption coefficient is a crucial determinant of a material’s appropriateness PV and optoelectronic applications. Figure 2d illustrates that Na_2_AuGaBr_6_ displays its initial absorption onset at 1.54 eV, which closely aligns with its electronic bandgap, so validating its responsiveness to visible photon energy. This beginning underscores its promise as an effective light-harvesting material. Improving absorption in this area could markedly enhance device performance, rendering it competitive with other advanced perovskite absorbers in the solar cell industry.

The reflectivity spectrum demonstrated in Fig. 2e, elucidates the material’s interaction with incident light across various energy ranges. The reflectance varies significantly throughout the photon energy ranges of 0-17.5 eV, exhibiting a notable rise in the visible spectrum. This behavior highlights the significance of surface shape, crystal orientation, and composition in determining optical response^41^. The increased reflectivity in the visible spectrum is significant, as it directly affects light management in photovoltaic systems, potentially enhancing external quantum efficiency when tuned.

The electron energy-loss function (L(ω)) was examined, as it offers insights into the energy dissipation of rapid electrons moving across the dielectric medium under illumination. This function is represented mathematically in Eq. (4).

4 $$\:\mathrm{L}\:\left({\upomega\:}\right)=\mathrm{j}\:(\:-\:\frac{1}{{\upepsilon\:}\:\left({\upomega\:}\right)}\:)$$

Figure 2f presents a spectrum exhibit prominent peaks between the ranges of 10.5–16 eV, with the apex peak attaining a value of 2.43 at 15.7 eV. These peaks correspond to plasmon resonance frequencies, where collective electron oscillations govern the optical response^42^. Significantly, no distinct peaks are detected below 2.15 eV, affirming that Na_2_AuGaBr_6_ is exceptionally proficient in absorbing visible and near-infrared light while reducing electron energy losses in this vital spectral region. The results collectively indicate that Na_2_AuGaBr_6_ has a favorable combination of an appropriate absorption edge, elevated dielectric constant, and minimal optical losses within the visible spectrum. These characteristics strongly indicate their promise as a resilient and efficient material for photovoltaic and optoelectronic applications, where effective light absorption and reduced recombination losses are essential for optimal performance.

### SCAPS 1D result

#### Architectural design of the solar cell device

**Fig. 3:**
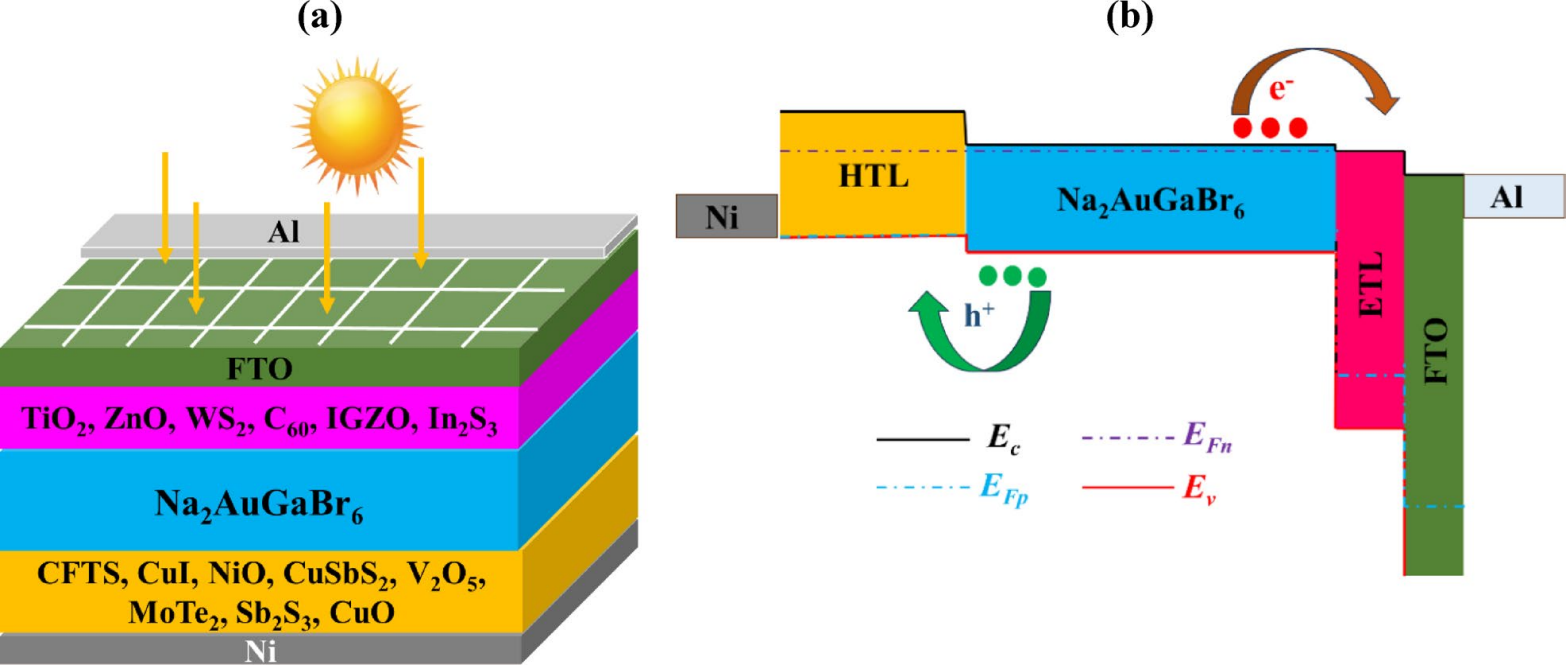
(**a**) Schematic diagram and (**b**) EBA of Al/FTO/ETL/Na_2_AuGaBr_6_/HTL/Ni solar cell.

This research simulated a DPSC utilizing Na_2_AuGaBr_6_ with an n-i-p planar heterojunction configuration. Figure 3a,b illustrates the device structure and energy band alignment (EBA), which includes ETLs, the perovskite absorber, and HTLs in the n, i, and p regions. Upon illumination, excitons are generated in the perovskite and subsequently diffuse into neighboring layers. At the n–i and i–p junctions, excitons dissociate, facilitating the movement of electrons to the n-layer and holes to the p-layer. The inherent electric field promotes excitement, dissociation and charge mobility^43^. To optimize this process, the simulation also evaluated various ETL candidates including TiO_2_, ZnO, WS_2_, C_60_, IGZO, and In_2_S_3_ along with Al and Ni as the front and back contact, FTO as the transparent front contact, and CFTS, CuI, NiO, CuSbS_2_, V_2_O_5_, MoTe_2_, Sb_2_S_3_, and CuO as possible HTLs. Na_2_AuGaBr_6_ was utilized as the absorber layer in the device. The device simulation was conducted with SCAPS-1D, utilizing input parameters derived from previous research as shown in Tables 1 and 2. The interface features for the complete device structure are delineated in Table 3. The model designated Al and Ni as the anode and cathode, respectively, employing a drift velocity (electrons and holes) of 10^7^ cm/s. Simulations incorporated variables such as doping concentrations, layer thickness, trap densities, and temperature, while assessing critical performance metrics like J-V curves, QE, band structure, and recombination profiles, under conventional AM 1.5G illumination (100 mW/cm^2^) at 300 K.

**Table 1:** Simulation variables of FTO, ETLs, and absorber for SCAPS-1D modelling^11,44,45^.

Parameter (unit)	FTO	TiO_2_	ZnO	WS_2_	C_60_	IGZO	In_2_S_3_	Na_2_AuGaBr_6_
N (µm)	0.1	0.05	0.05	0.05	0.05	0.05	0.05	1
E_g_ (eV)	3.9	3.2	3.3	1.8	1.7	3.05	2.1	1.54
χ (eV)	4	4	4	3.9	3.9	4.16	4	3.83
ε	9	9	9	13.6	4.2	10	13.5	30.17
N_V_ (cm^− 3^)	1.8 × 10^19^	1.8 × 10^19^	1.8 × 10^19^	1.8 × 10^19^	8 × 10^19^	5 × 10^18^	4 × 10^13^	2.11 × 10^19^
N_C_ (cm^− 3^)	2.2 × 10^18^	2.2 × 10^18^	3.7 × 10^18^	2.2 × 10^18^	8 × 10^19^	5 × 10^18^	1.8 × 10^19^	9.46 × 10^18^
µ_n_ (cm^2^V^−1^s^− 1^)	100	20	100	100	0.08	15	400	80
µ_h_ (cm^2^V^−1^s^− 1^)	25	10	25	100	0.0035	0.1	210	25
N_A_ (cm^− 3^)	0	0	0	0	0	0	0	1 × 10^18^
N_D_ (cm^− 3^)	1 × 10^19^	1 × 10^18^	1 × 10^18^	1 × 10^18^	1 × 10^18^	1 × 10^18^	1 × 10^18^	0
N_t_ (cm^− 3^)	1 × 10^15^	1 × 10^15^	1 × 10^15^	1 × 10^15^	1 × 10^15^	1 × 10^15^	1 × 10^15^	1 × 10^14^

**Table 2:** The input variables for HTL layers^46,47^.

Parameter (unit)	CFTS	CuI	NiO	CuSbS_2_	V_2_O_5_	MoTe_2_	Sb_2_S_3_	CuO
N (µm)	0.1	0.1	0.1	0.1	0.1	0.1	0.1	0.1
E_g_ (eV)	1.87	3.1	3.8	1.58	2.2	1.29	1.7	1.51
χ (eV)	3.3	2.1	1.46	4.2	3.4	4.2	3.7	4.07
ε	9	6.5	10.7	14.6	8	13	7.08	18.1
N_C_ (cm^− 3^)	2.2 × 10^18^	2.8 × 10^19^	2.8 × 10^19^	2 × 10^18^	9.2 × 10^19^	4 × 10^16^	2 × 10^19^	2.2 × 10^19^
N_V_ (cm^− 3^)	2.8 × 10^19^	1 × 10^19^	1 × 10^19^	1 × 10^19^	5 × 10^19^	3 × 10^18^	1 × 10^19^	5.5 × 10^20^
N_A_ (cm^− 3^)	1 × 10^20^	1 × 10^20^	1 × 10^20^	1 × 10^18^	1 × 10^20^	1 × 10^19^	1 × 10^18^	1 × 10^20^
µ_n_ (cm^2^V^−1^s^− 1^)	21.98	100	100	49	150	110	9.8	100
µ_h_ (cm^2^V^−1^s^− 1^)	21.98	43.9	43.9	49	100	426	10	0.1
N_t_ (cm^− 3^)	1 × 10^15^	1 × 10^15^	1 × 10^15^	1 × 10^15^	1 × 10^15^	1 × 10^15^	1 × 10^15^	1 × 10^15^
N_D_ (cm^− 3^)	0	0	0	0	0	0	0	0

**Table 3:** Characteristics of interfacial defects.

Parameter	Na_2_AuGaBr_6_/ETL	HTL/Na_2_AuGaBr_6_
Type of defect	Neutral	
E_r_ (eV)	0.6	
Energetic distribution	Single	
σ_h_ /σ_e_ (cm^2^)	1 × 10^− 19^	
Total density (cm^− 2^)	1 × 10^12^	

#### Effect of ETL and HTL variation

**Fig. 4:**
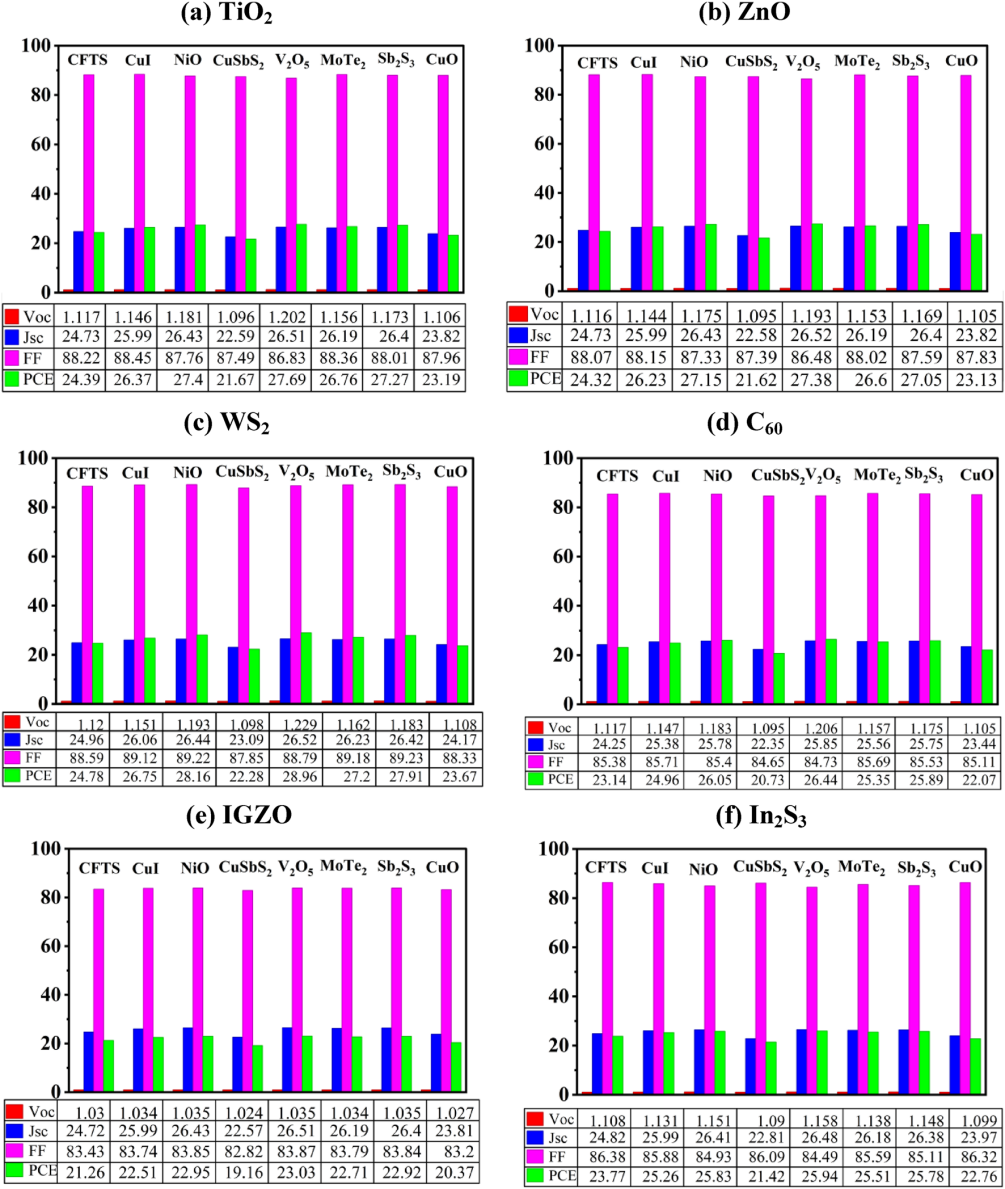
Influence of various HTLs in conjunction with varied ETLs, specifically (**a**) TiO_2_, (**b**) ZnO, (**c**) WS_2_, (**d**) C_60_, (**e**) IGZO, and (**f**) In_2_S_3_, on the PV performance of Na_2_AuGaBr_6_ absorber-based solar cells.

The different combinations of eight HTLs and six ETLs were tested to maximize the performance of the Na_2_AuGaBr_6_-based DPSC. A total of 48 Na_2_AuGaBr_6_-based device configurations were systematically simulated to investigate the impact of six ETLs and eight HTLs on PV performance, as detailed in Table S1 (Supporting Information). Table 4 enumerates the six most effective configurations of the PSCs, yielding the highest PCE with various ETLs beside the best HTL. Figure 4a–f illustrates that the configuration FTO/WS_2_/Na_2_AuGaBr_6_/V_2_O_5_/Ni attained the maximum PCE of 28.96%, accompanied by a J_SC_ of 26.52 mA/cm^2^, an FF of 88.79%, and a V_OC_ of 1.229 V. It also demonstrates that ETLs such as TiO_2_, ZnO, WS_2_, and In_2_S_3_ surpassed others like C_60_ and IGZO in PCE, attributable to their advantageous band alignment. When utilized WS_2_ as the ETL, HTLs such as CuI, NiO, V_2_O_5_, MoTe_2_, and Sb_2_S_3_ attained impressive PCEs of 26.75%, 28.16%, 28.96%, 27.2%, and 27.91%, respectively, but HTLs including CFTS, Cu_S_bS_2_, and CuO resulted in diminished PCEs (below 25%). Among the six ETLs analyzed with eight different HTLs, V_2_O_5_ consistently demonstrated higher PCE. Our experiments indicated that inorganic HTLs typically surpassed organic counterparts, with some exceptions. This is mainly due to the superior stability, elevated transparency, and improved band alignment of the inorganic HTLs. The V_2_O_5_ HTL, demonstrated exceptional performance for each ETL combination because of its optimal atomic size, superior crystalline structure, and light-absorbing capacity^48^. The optimally improved architecture for the Na_2_AuGaBr_6_-based DPSC, utilizing V_2_O_5_ as HTL and WS_2_ as ETL.

**Table 4:** Comparative analysis of various ETLs with the optimal HTL.

Structures	V_OC_ (V)	J_SC_ (mA/cm^2^)	FF (%)	PCE (%)
FTO/TiO_2_/Na_2_AuGaBr_6_/V_2_O_5_/Ni	1.202	26.51	86.83	27.69
FTO/ZnO/Na_2_AuGaBr_6_/V_2_O_5_/Ni	1.193	26.52	86.48	27.38
FTO/WS_2_/Na_2_AuGaBr_6_/V_2_O_5_/Ni	1.229	26.52	88.79	28.96
FTO/C_60_/Na_2_AuGaBr_6_/V_2_O_5_/Ni	1.206	25.85	84.73	26.44
FTO/IGZO/Na_2_AuGaBr_6_/V_2_O_5_/Ni	1.035	26.51	83.87	23.03
FTO/In_2_S_3_/Na_2_AuGaBr_6_/V_2_O_5_/Ni	1.158	26.48	84.49	25.94

#### Influence of metal work function on the efficiency of solar cells

**Fig. 5:**
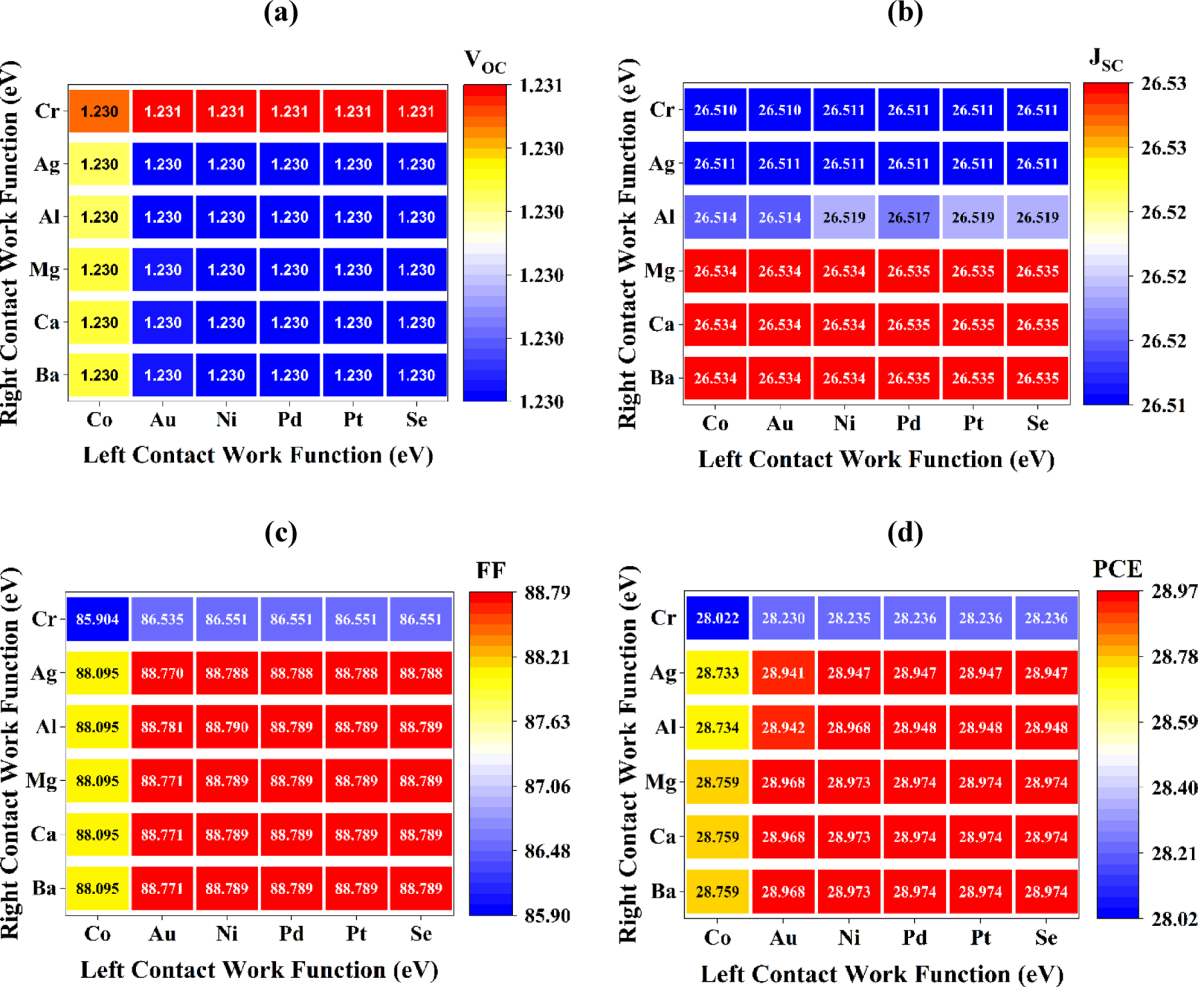
Examination of metal contact impacts on (**a**) V_OC_, (**b**) J_SC_, (**c**) FF, and (**d**) PCE in the optimal configuration.

The work function of metal contacts is crucial in determining the efficiency and charge transport characteristics of PSCs. The appropriate selection and engineering of contact metals greatly affect band alignment, carrier extraction, and recombination dynamics, therefore influencing overall device performance. The left (back) contact must effectively gather holes and minimize resistance losses to optimize current flow and decrease energy dissipation. Inadequately designed connections can elevate series resistance, restrict charge transport, and cause shading effects, thereby diminishing light absorption and the active area for carrier collection^49^. In contrast, a well-designed interface enhances charge extraction efficiency, reduces recombination losses, and elevates critical performance metrics such as FF and V_OC_^50^. Figure 5a–d illustrates the PV properties of the WS_2_/Na_2_AuGaBr_6_/V_2_O_5_ device employing various left (Co, Ni, Au, Pt, Pd, and Se) and right (Ca, Ba, Mg, Ag, Al, and Cr) metal contacts to evaluate their impact on photovoltaic performance. The metal work functions of these metals are compiled in Table S2. Nickel (Ni) and aluminum (Al) exhibited the most advantageous properties owing to their complementing work functions. Nickel, utilized as the back contact, exhibits a high work function and robust temperature and chemical stability, facilitating efficient hole extraction^51^. Conversely, Al, acting as the primary contact, possesses a low work function, superior conductivity, and high reflectivity, hence improving electron collection. The optimized Al/FTO/WS_2_/Na_2_AuGaBr_6_/V_2_O_5_/Ni combination attained a remarkable PCE of 28.96%, J_SC_ of 26.52 mA/cm^2^, FF of 88.79%, and V_OC_ of 1.229 V, underscoring the significance of metal work function modulation in enhancing device performance.

#### Band structure

**Fig. 6:**
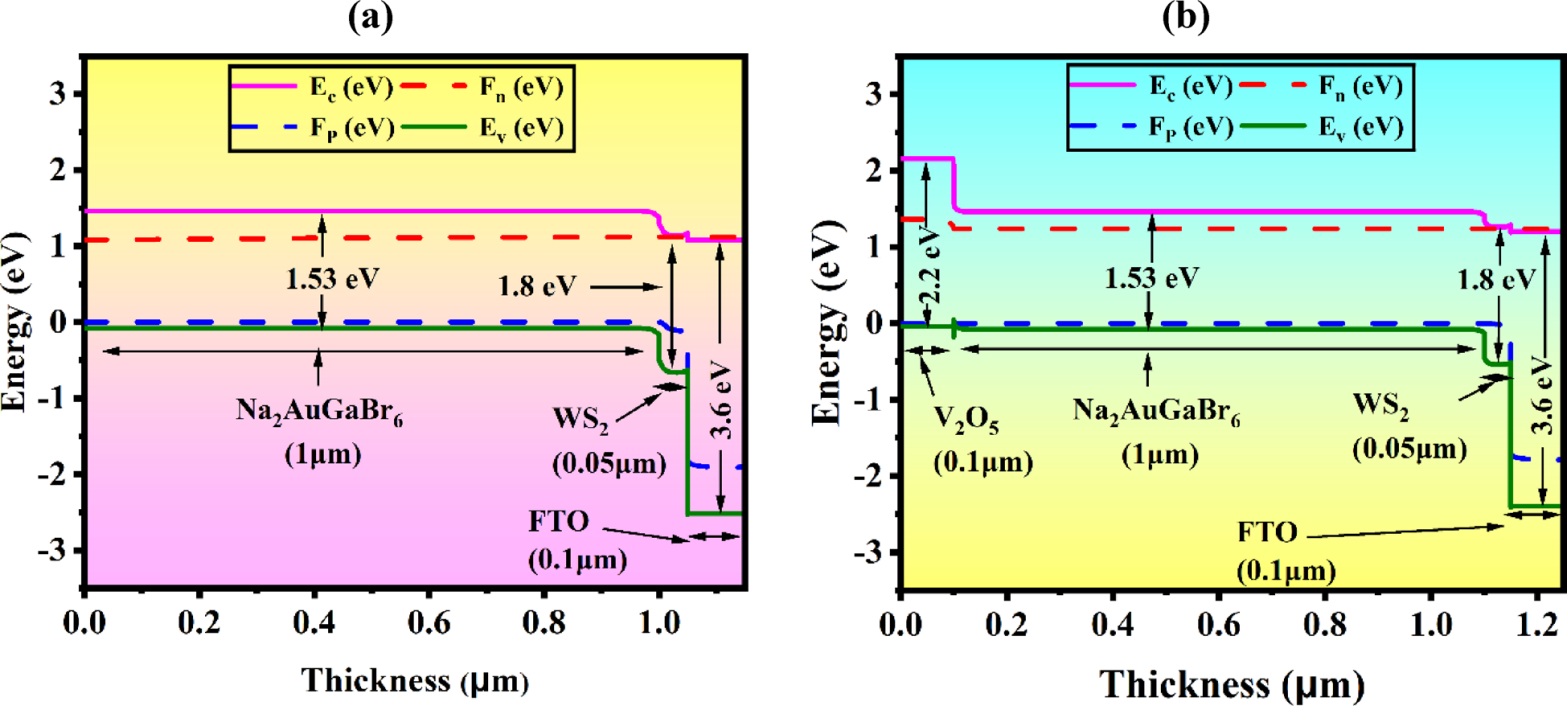
(**a**) Al/FTO/WS_2_/Na_2_AuGaBr_6_/Ni and (**b**) Al/FTO/WS_2_/Na_2_AuGaBr_6_/V_2_O_5_/Ni cells energy band diagrams.

The energy band diagrams of Al/FTO/WS_2_/Na_2_AuGaBr_6_/Ni and Al/FTO/WS_2_/Na_2_AuGaBr_6_/V_2_O_5_/Ni cells, illustrated in Fig. 6a,b, demonstrate the influence of HTL such as V_2_O_5_ on carrier dynamics and energy alignment. The architecture incorporates V_2_O_5_ as a heavily doped p⁺-type HTL and WS_2_ as an n-type ETL, resulting in a p⁺–p–n–n⁺ configuration. WS_2_ is preferred due to its CB alignment with diverse absorbers, enabling effective electron extraction from Na_2_AuGaBr_6_, although its reduced CB density of states (DOS) relative to V_2_O_5_^52,53^. Concurrently, the substantial valence band (VB) of V_2_O_5_ offers enhanced hole transport efficiency, attributable to its high VB density of states. Although V_2_O_5_ exhibits superior electron mobility compared to WS_2_, its function as a HTL is validated by its capacity to obstruct electrons and for efficient hole transfer alignment. EBA is crucial for device performance. V_2_O_5_ creates a significant conduction band offset (CBO) at the rear interface, significantly obstructing electron migration to the Ni back contact and thus diminishing recombination. In contrast, a little CBO between Na_2_AuGaBr_6_ and WS_2_ facilitates electron transit to the front contact. A substantial valence band offset (VBO) inhibits hole leakage, hence augmenting selectivity. Notwithstanding its advantages, a Schottky barrier may form at the Na_2_AuGaBr_6_/V_2_O_5_ contact due to VB misalignment, which might impede hole transport and facilitate recombination^54^. This problem can be alleviated using interface engineering techniques, such as the use of intermediary layers or surface passivation, to reduce barrier height and improve carrier mobility. To achieve efficient charge separation, it is essential to improve the alignments of both the valence and conduction bands, facilitating hole transport towards V_2_O_5_ and electron movement towards WS_2_. An expanded bandgap in the ETL diminishes photon absorption losses and enhances J_SC_.

#### Analyzing absorber thickness and carrier concentration effects on PV performance

**Fig. 7:**
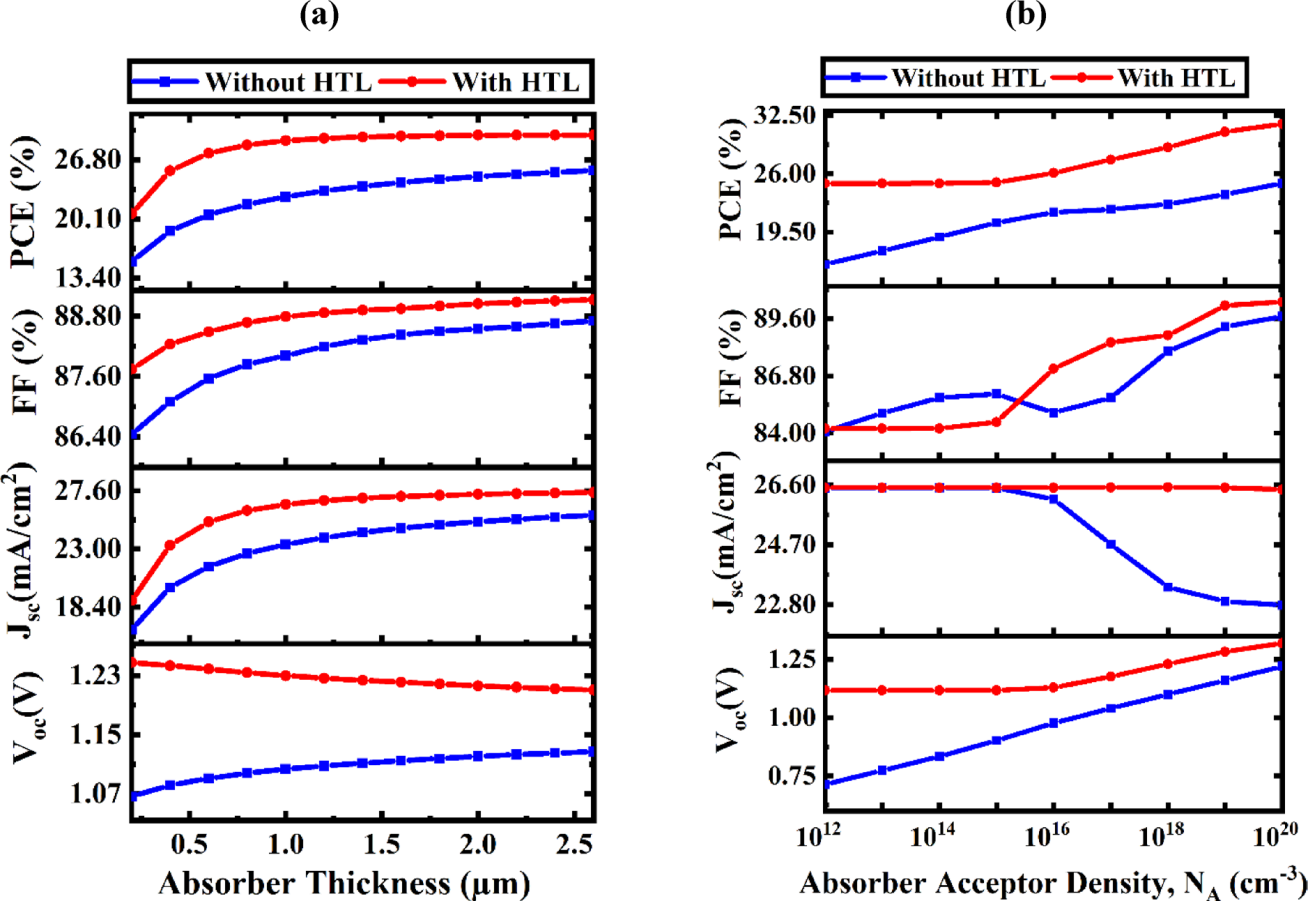
Study of the impact of (**a**) absorber layer thickness and (**b**) N_A_ in Na_2_AuGaBr_6_ with and without the HTL.

Figure 7a depicts the response of PV module efficiency characteristics as the absorber thickness varies from 0.20 μm to 2.6 μm. Increasing the absorber depth while incorporating a V_2_O_5_ HTL significantly enhances device performance, especially in V_OC_, across various thicknesses. In the absence of HTL, a pronounced rise in V_OC_ is noted below 1.0 μm, with further enhancements occurring thereafter. This tendency corresponds with findings documented in previous studies^55,56^. In the absence of HTL, PCE increases from 15.27% to 25.59% with greater thickness. The integration of the HTL significantly enhances efficiency, increasing from 20.71% to 29.59% within the same thickness range. The FF demonstrates a gradual increase as the active layer thickness falls between 0.20 μm and 1.6 μm, ultimately stabilizing at approximately 2.6 μm. In HTL-enhanced cells, the J_SC_ first increases proportionally with thickness up to 1.0 μm, subsequently continuing to rise more gradually, attaining values of 26.520 and 27.451 mA/cm^2^. Conversely, in the absence of HTL, J_SC_ increases linearly to 1.6 μm, subsequently rising slightly to 24.645 and 25.664 mA/cm^2^. The improvements in J_SC_ are primarily attributable to enhanced photon absorption at extended wavelengths in both the absorber and V_2_O_5_ layers, resulting in an increased generation of electron-hole pairs (EHPs). However, as the absorber exceeds a depth of 2.6 μm, the recombination of photogenerated carriers increases, leading to reduced efficiency gains.

Figure 7b illustrates that in the absence of HTL, the PCE significantly rises from 15.27 to 25.59% when N_A_ surpasses 10^18^ cm^− 3^. With the integration of the HTL, the PCE enhances from 20.71 to 29.59% as doping escalates from 10^10^ to 10^20^ cm^− 3^. Although the FF experiences a modest increase (from 87.74 to 89.12%), a minor reduction in V_OC_ (from 1.247 V to 1.209 V) is noted under these conditions. Notwithstanding this decline, device performance stays consistent owing to increased hole density, which fortifies the internal electric field and enhances both V_OC_ and FF. The thickness of the Na_2_AuGaBr_6_ absorber layer is essential for optimizing solar spectrum absorption. As light penetrates further, a denser layer facilitates the generation of additional EHPs.

Nevertheless, thicker layers augment the travel distance for charge carriers, hence elevating the danger of recombination losses. Consequently, determining an ideal thickness is essential for attaining efficient charge extraction. Manufacturing methods and economic concerns can influence the determination of optimal thickness^57,58^. Thicker layers may necessitate more intricate deposition methods, thus increasing production costs. For Na_2_AuGaBr_6_-based PV devices, it is advisable to utilize an absorber depth of roughly 1000 nm in conjunction with a doping concentration of 10^18^ cm^− 3^, while balancing the associated trade-offs.

#### Impact of ETL thickness and doping concentration (N_D_) on performance metrics

**Fig. 8:**
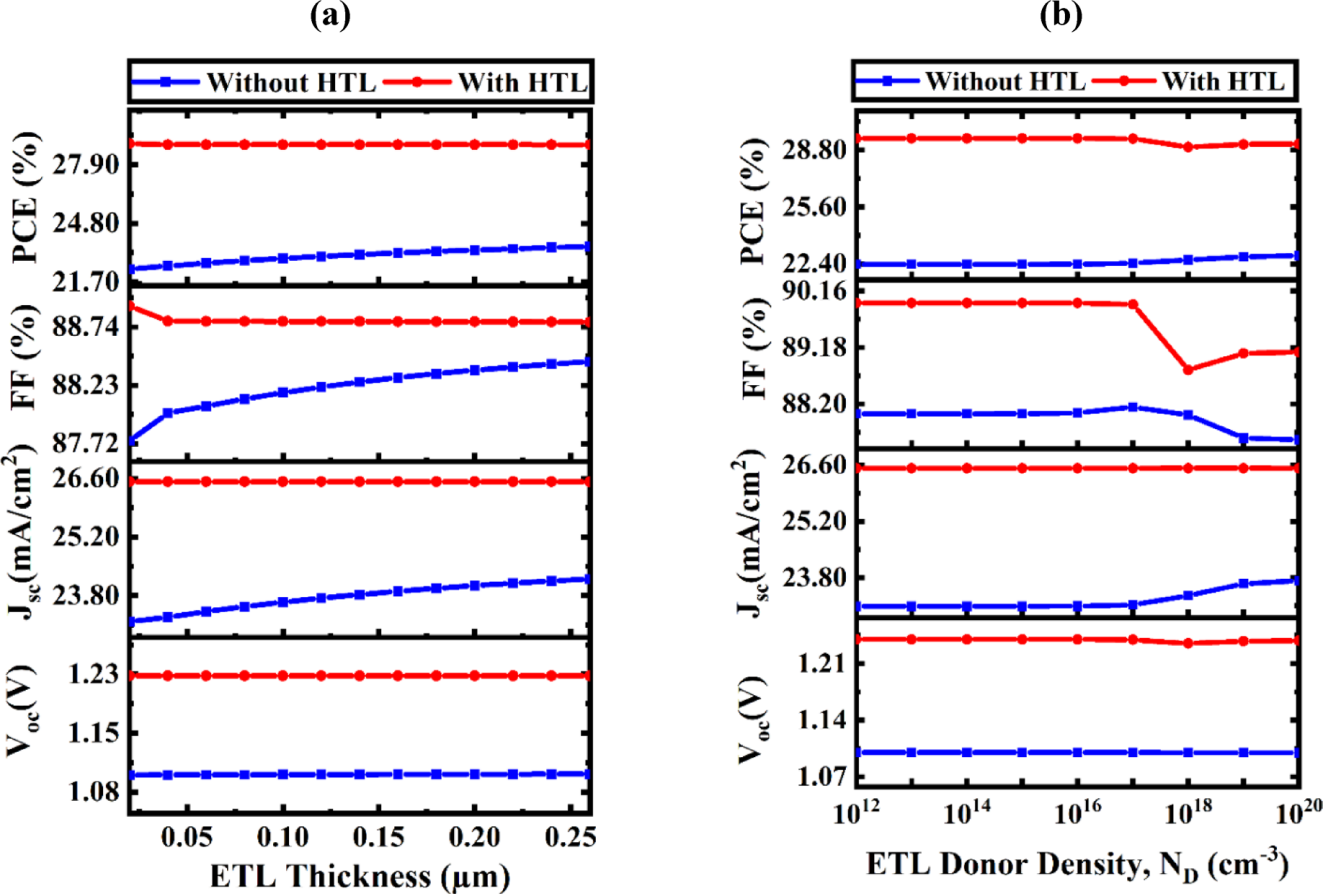
Enhancement of SC efficacy: the influence of WS_2_ (**a**) ETL thickness and (**b**) doping concentration.

The performance of PV systems is significantly impacted by the thickness of the ETL, as shown in Fig. 8a. ETL improves device stability and efficiency by serving as a bridge between the absorber and the electrode and facilitating charge carrier transfer. Light absorption, carrier recombination, and interfacial resistance are among the properties that are impacted by the depth of this layer^59^. ETL layers effectively transport incoming photons to the Na_2_AuGaBr_6_ absorber when they are thin enough and have an enlarged bandgap, which improves photon absorption and reduces optical losses. These thin ETLs maximize the SC’s overall photo response by letting almost all incident light through. Remarkably, the V_OC_ was not much affected by changes in ETL thickness. While devices with HTL stayed constant at 1.229 V, those without HTL- V_OC_ remain stable at about 1.10 V. Likewise, there was stability in the J_SC_. J_SC_ continuously monitored 26.521 mA/cm^2^ in cells with HTL, while devices without an HTL showed a slight increase, changes from 23.176 to 24.197 mA/cm^2^. This slight increase demonstrates that ETL thickness has little impact on J_SC_ within the ideal range. ETL is sufficiently thick to stop charge leakage without impeding electron transit, which is the obvious explanation for this stability. Recombination is so inhibited, and carrier extraction continues to be effective. These features of ETL designs provide dependable performance at various thicknesses. There was also a minor change in FF. While device with HTL showed a steady FF of 88.78%, devices lacking HTL showed a modest increase in FF from 87.74 to 88.43%. The PCE showed a similar trend. PCE increased from 22.37 to 23.56% for HTL-free cells while staying constant at 28.95% for devices that included HTL. These modifications imply that ETL thickness has no effect on PV performance metrics within the assessed range. An ETL thickness of 50 nm is considered ideal for achieving an appropriate balance between manufacturing viability and performance. It is difficult to produce layers thinner than this threshold using conventional production methods.

Figure 8b analyses the influence of N_D_ on the PV characteristics of ETL. Doping in ETL layer is a prevalent method to improve the conductivity and EBA between the absorber layer and the electrode. The N_D_ influences various aspects of PV devices, such as bandgap, conductivity, and carrier concentration^60^. For the WS_2_ ETL, the performance metrics exhibited little fluctuations as N_D_ varied from 10^12^ to 10^20^ cm^− 3^. As N_D_ increased, the V_OC_, PCE, FF, and J_SC_ demonstrated negligible variations: from 1.1006 V to 1.1003 V, 22.37% to 22.86%, 88.03% to 87.58%, and 23.0909 mA/cm^2^ to 23.7246 mA/cm^2^ for the device lacking HTL; from 29.45% to 29.13%, 89.94% to 89.10%, 1.2346 V to 1.2331 V, and 26.5200 mA/cm^2^ to 26.5174 mA/cm^2^ for the device incorporating V_2_O_5_ HTL. The data suggests that fluctuations in doping concentration within the examined range have a minimal impact on the PV performance of the ETL.

#### Parametric analysis of HTL thickness and N_A_ on solar device efficiency

**Fig. 9:**
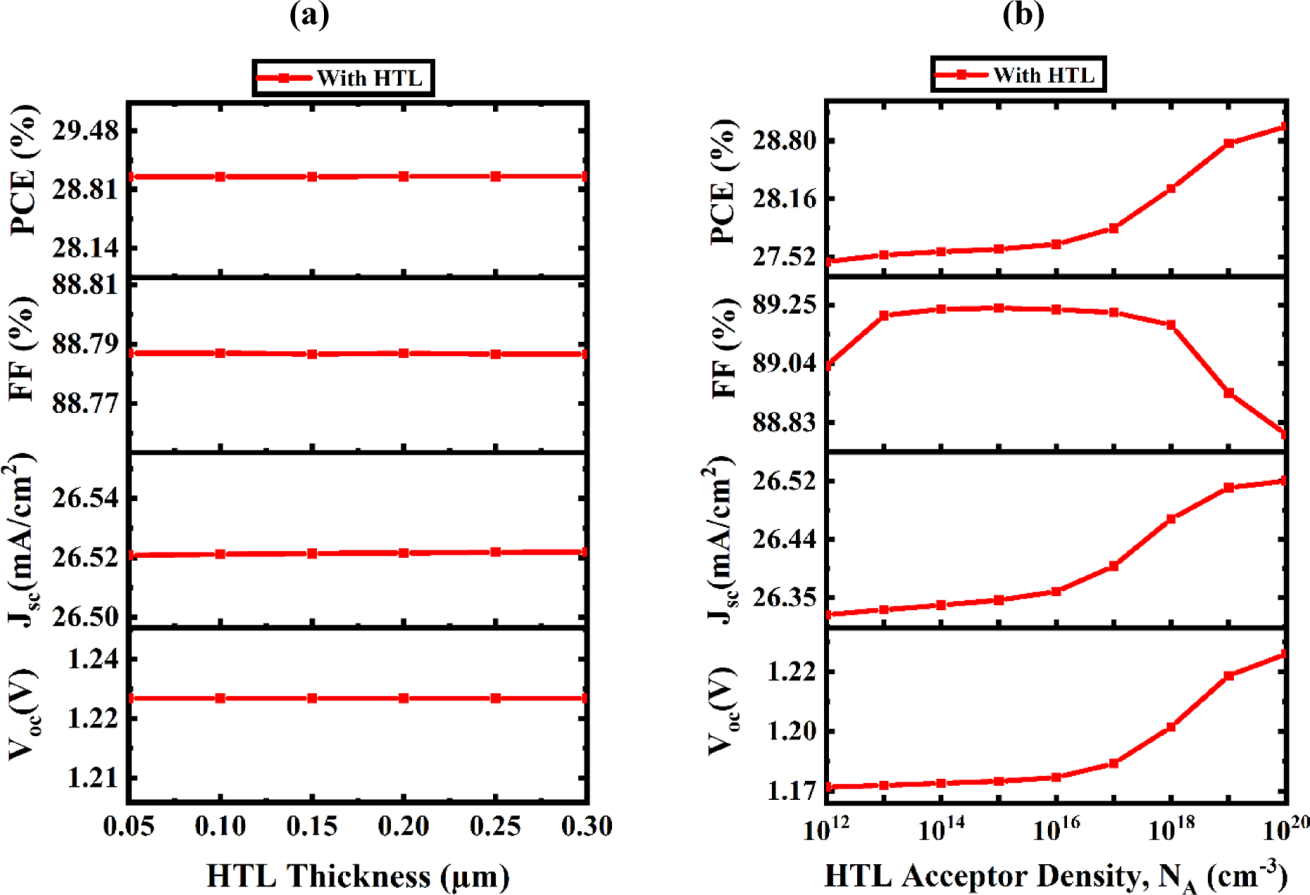
Performance analysis of PV devices considering variations in HTL (**a**) thickness and (**b**) doping concentrations.

The integration of a HTL decreases surface recombination velocity and establishes a robust electric field, hence enhancing SC efficiency. The incorporation of a V_2_O_5_ HTL boosts carrier mobility and reduces recombination in Na_2_AuGaBr_6_-based PSCs by optimizing electron collecting at the rear contact. Figure 9a,b demonstrate the influence of the HTL on SC performance via alterations in carrier density and thickness. Figure 9a illustrates the variation of HTL thickness from 0.05 μm to 0.3 μm. The V_OC_ and FF values stay largely constant, although the PCE and J_SC_ exhibit slight fluctuations, varying from 28.9578 to 28.9588% and from 26.5206 to 26.5216 mA/cm^2^, respectively. The higher series resistance of thicker HTL layers is responsible for the slight variations in performance^61^. FF and PCE are significantly impacted by the resistance increase, which is directly correlated with the HTL layer’s thickness. Significantly, no substantial difference in the PV performance parameters is detected with increasing V_2_O_5_ thickness, demonstrating that manipulation of the V_2_O_5_ layer has a minimal impact on the overall device performance. This thickness-insensitive behavior aligns with previously reported research that exhibit analogous tendencies in charge transport layer optimization^62,63^.

Thus, we have determined 50 nm to be the optimal thickness for HTL, optimizing efficiency while minimizing production costs. Enhancing the N_A_ in the HTL is a prevalent method to improve the efficiency of PV systems^64^. This alteration affects several attributes, including alignment of band structure and conductivity, both essential for optimizing device performance. Figure 9b depicts the impact of altering carrier concentration in HTL, ranging from 10^12^ to 10^20^ cm^− 3^. The increase in doping density within the V_2_O_5_ layer results in a proportional enhancement of V_OC_, J_SC_, and PCE. The PCE rises from 27.464% to 28.958%. To enhance PV performance by enabling effective charge carrier extraction and transport, the HTL must have an N_A_ level of at least 10^20^ cm^− 3^.

#### Assessment of SC performance under varying Na_2_AuGaBr_6_ layer thickness and defect density (N_t_) conditions

**Fig. 10:**
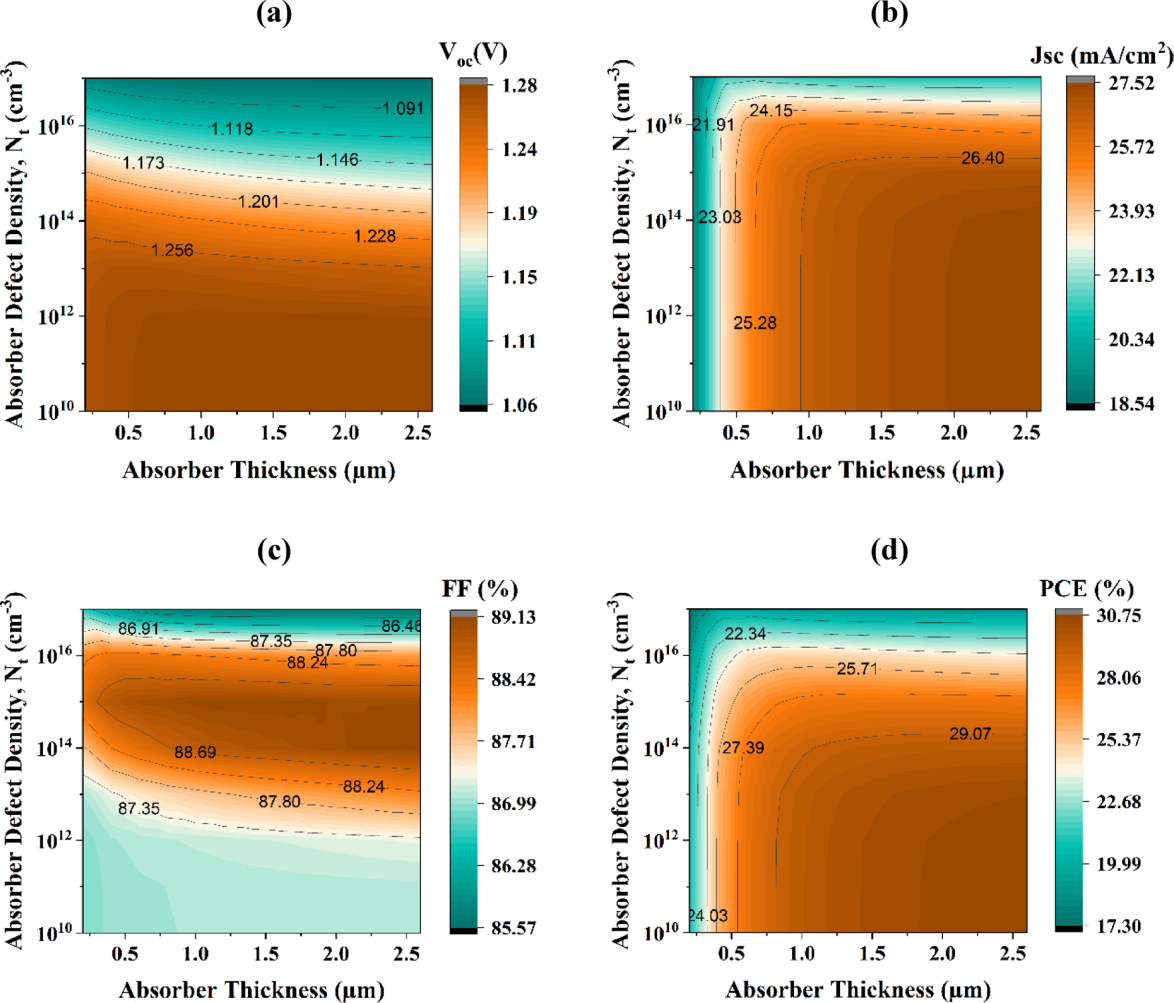
Comparative analysis of variations in acceptor and bulk defect density in Na_2_AuGaBr_6_ with V_2_O_5_ HTL on SC performance indicators.

Figure 10a–d illustrates the influence of defect density (N_t_), varied from 10^10^ to 10^17^ cm^− 3^, and absorber layer thickness, ranging from 0.2 to 2.6 μm, on the PV performance characteristics of the device. Examining a broad spectrum of defect concentrations facilitates a comprehensive assessment of defect tolerance, recombination kinetics, and carrier lifetime constraints, while alterations in absorber thickness elucidate the balance between optical absorption and charge transport^65^. This thorough parameter-space analysis enables the development of optimal device configurations and evaluates performance stability under both ideal and defect-laden substantial conditions. In Fig. 10a, the V_OC_ exhibits considerable stability across varying absorber thicknesses. V_OC_ significantly decreases from 1.2794 V to 1.0631 V when N_t_ fluctuates between 10^10^ and 10^17^ cm^− 3^, highlighting the negative effect of defect states on device efficacy. The J_SC_ reaches a maximum of 27.503 mA/cm^2^ when the absorber layer depth is 1.6 μm and N_t_ is maintained below 10^12^ cm^− 3^, as illustrated in Fig. 10b. As the thickness diminishes from 2.6 μm to 0.2 μm under low N_t_ circumstances (< 10^14^ cm^− 3^), J_SC_ declines by 8.9518 mA/cm^2^. This decrease is ascribed to a reduced charge carrier diffusion length and heightened recombination activity, both of which impede current generation efficiency^66^.

Figure 10c indicates that the FF reaches its maximum value of 89.09% at a N_t_ of 10^15^ cm^− 3^. However, when N_t_ surpasses 10^15^ cm^− 3^, the FF declines significantly to 85.57%. This decline is presumably due to increased recombination, which reduces the number of charge carriers obtainable for power production. It is evident that augmenting N_t_ adversely impacts the overall efficiency of the gadget. Minimizing N_t_ is essential, as decreased defect densities provide longer carrier diffusion lengths and diminish recombination losses, so improving PV efficiency. Optimal outcomes are attained when the absorber demonstrates both a minimal trap density and a maximal carrier concentration. A maximum PCE of 28.96% was observed (Fig. 10d) at an absorber thickness of 1.0 μm and N_t_ of 10^14^ cm^− 3^, which is consistent with reports on similar double perovskite materials^67,68^. Under these optimal conditions, the device exhibits a V_OC_ of 1.229 V, a J_SC_ of 26.52 mA/cm^2^, and an FF of 88.79%.

#### Optimization of interface defect densities (N_tf_) for improved PV performance

**Fig. 11:**
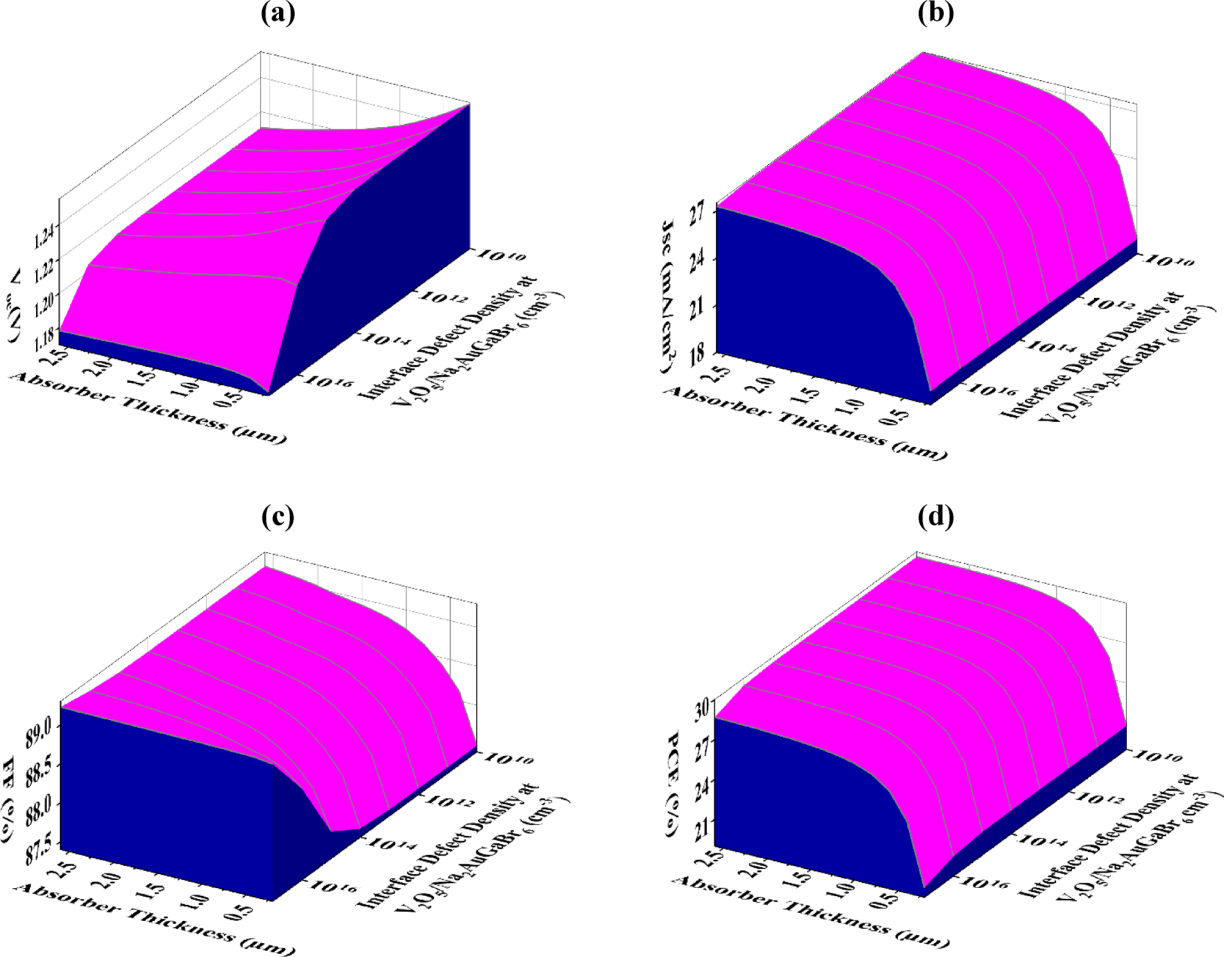
Impact of variations in absorber thickness and N_tf_ at the V_2_O_5_/Na_2_AuGaBr_6_ interface on PV efficacy: (**a**) V_OC_, (**b**) J_SC_, (**c**) FF, and (**d**) PCE.

**Fig. 12:**
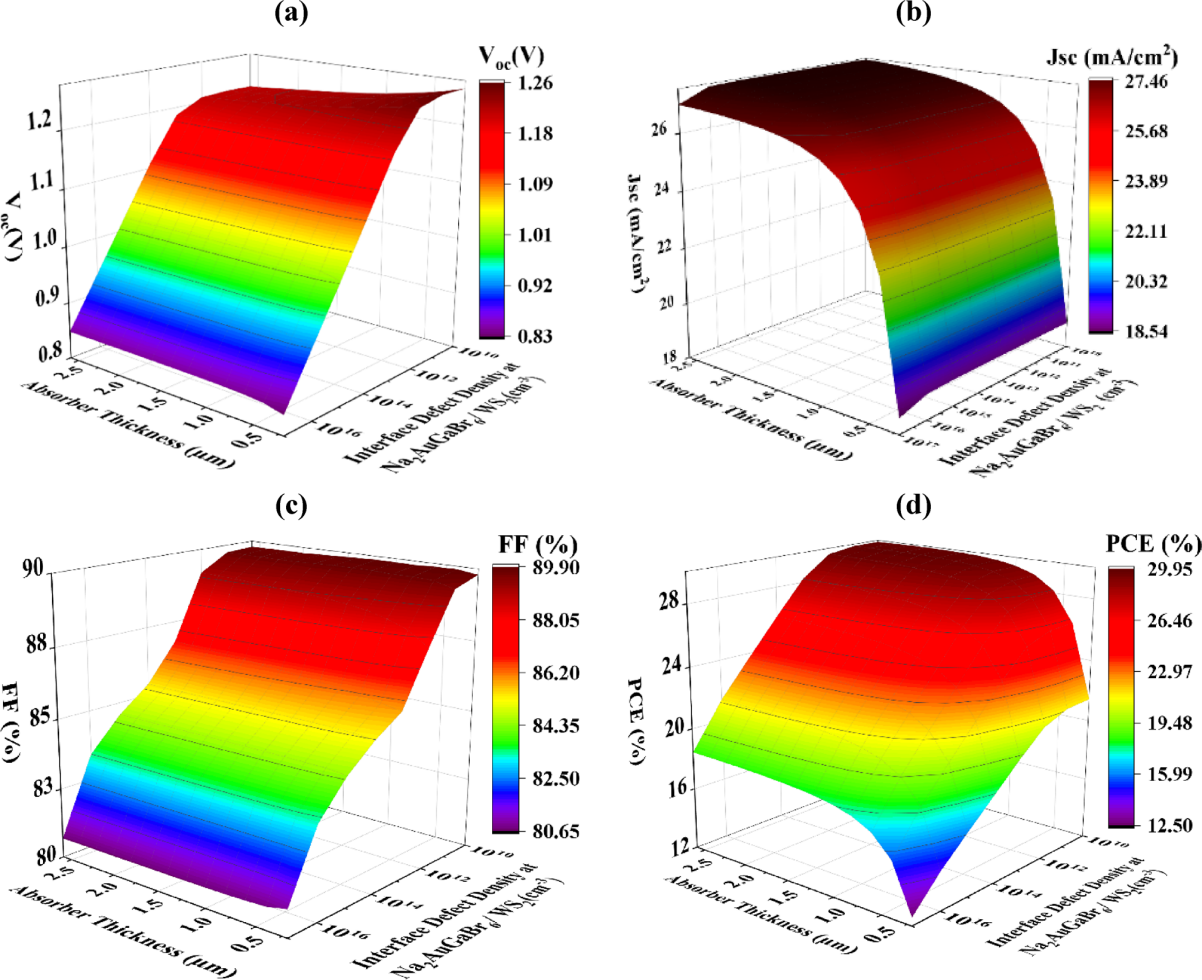
Combined impact of absorber layer depth and Na_2_AuGaBr_6_/WS_2_ interface N_tf_ on PV performance metrics.

Defects at the interface layer in SCs substantially elevate recombination losses, resulting in a quantifiable reduction in device performance. The interface flaws generate trap states that impede charge carrier movement and collection, hence diminishing overall efficiency and hastening deterioration, which jeopardizes long-term device stability^69^. This study investigates the impact of N_tf_ and absorber layer depth on the performance of two significant interfaces: V_2_O_5_/Na_2_AuGaBr_6_ and Na_2_AuGaBr_6_/WS_2_. The parameters were adjusted throughout a broad spectrum: N_tf_ ranged from 10^10^ to 10^17^ cm^− 2^ and absorber thickness varied from 0.2 μm to 2.6 μm. Figures 11a–d and 12a––d illustrate that an increase in N_tf_ results in a significant decline in the electrical properties of both surfaces.

Figure 11a–d presents performance metrics for the V_2_O_5_/Na_2_AuGaBr_6_ interface. As N_tf_ rises, PCE diminishes from 29.61% to 19.70%, FF reduces from 89.22% to 87.47%, J_SC_ lowers from 27.45 mA/cm^2^ to 18.85 mA/cm^2^, and V_OC_ decreases from 1.2540 V to 1.1776 V. This pattern substantiates the adverse impact of elevated defect density at the contact. Figure 11a demonstrates that the peak V_OC_ of 1.25 V is achieved when N_tf_ is below 10^11^ cm^− 2^ and the absorber layer is below 1 μm. Beyond this level, V_OC_ demonstrates a significant decline, affirming the responsiveness of voltage output to interface trap density.

In Fig. 11c, FF stays largely stable until N_tf_ attains 10^11^ cm^− 2^, after which a minor increase is noted. This behavior indicates that intermediate levels of N_tf_ exert negligible influence on FF, whereas elevated concentrations commence to impact charge extraction dynamics. Figure 11b,d elucidate the ideal settings for optimizing J_SC_ and PCE. To attain a J_SC_ of 27.45 mA/cm^2^ and a PCE of 29.61%, the absorber depth must exceed 1 μm, and N_tf_ should remain below 10^15^ cm^− 2^. These values delineate a pragmatic threshold for interface quality in the construction of high-efficiency cells. Similar to earlier findings^70^, Fig. 12a–d depicts a notable decrease in performance for the Na_2_AuGaBr_6_/WS_2_ interface with an increase in N_tf_. The PCE declines significantly from 29.89% to 12.51%, and the F) decreases from 89.83% to 80.69%. Correspondingly, the J_SC_ diminishes from 27.45 mA/cm^2^ to 18.54 mA/cm^2^, and the V_OC_ declines from 1.2601 V to 0.8340 V. Figure 12a illustrates that the peak V_OC_ of 1.26 V is attained when N_tf_ is maintained below 10^12^ cm^− 2^, irrespective of absorber thickness. Once this threshold is surpassed, V_OC_ demonstrates a sharp decrease, signifying a pronounced sensitivity of the voltage to interface fault levels. Figure 12b indicates that the maximum J_SC_ values are observed when the absorber thickness surpasses 1 μm. Within this range, J_SC_ exhibits reasonable stability across different N_tf_ values, indicating that thicker absorber layers can offset moderate increases in interface defects. Figure 12c indicates that the optimal FF occurs when the absorber depth is under 1.0 μm and N_tf_ is below 10^11^ cm^− 2^. Exceeding these thresholds, the FF declines, possibly because of increased recombination at the interface that hinders charge extraction. Figure 12d indicates that the maximal PCE, surpassing 29%, is achieved with an absorber thickness between 1.0 and 1.4 μm, while maintaining N_tf_ at 10^12^ cm^− 2^.

This delineates the ideal equilibrium between thickness and interface quality for attaining high efficiency. The results indicate that the maximum tolerated N_tf_ for sustaining high performance is 10^15^ cm^− 2^ for the V_2_O_5_/Na_2_AuGaBr_6_ interface and 10^12^ cm^− 2^ for the Na_2_AuGaBr_6_/WS_2_ interface. In both instances, an absorber thickness of around 1 μm seems optimal. These findings align with the data published by Hassan et al.^71^, who noted analogous patterns in interface performance for both HTL/absorber and absorber/ETL contacts.

#### Effects of temperature fluctuations on PV device efficiency and stability

**Fig. 13:**
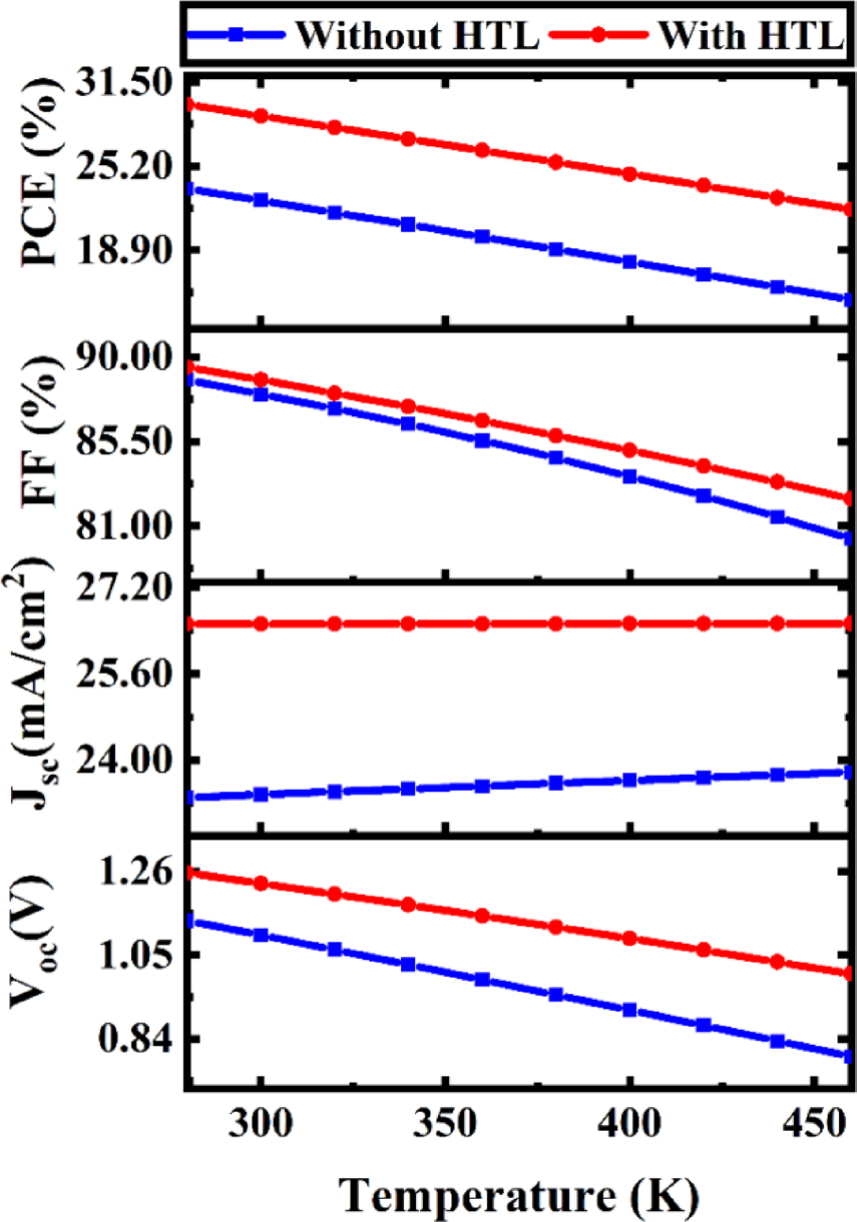
Temperature-dependent characteristics of PV cells.

Figure 13 depicts the fluctuation of essential photovoltaic parameters of the proposed Na_2_AuGaBr_6_-based solar cell in relation to operation temperature, assessed within the range of 280–460 K. Simulations dependent on temperature were performed utilizing a constant absorber thickness of 1.0 μm, with N_t_ of 10^14^ cm^− 3^, an ETL thickness of 0.05 μm at N_t_ of 10^15^ cm^− 3^, and HTL thickness of 0.1 μm at N_t_ of 10^15^ cm^− 3^. This temperature range was chosen to evaluate both standard operating settings and heightened thermal stress pertinent to actual solar applications. As temperature rises, thermal agitation intensifies, markedly affecting charge carrier dynamics within the device. Thermal agitation denotes the intensified vibrational movement of lattice atoms and charge carriers caused by elevated thermal energy. The heightened lattice vibration enhances carrier dispersion and raises the intrinsic carrier concentration, thereby increasing the reverse saturation current (J_0_)^72^. As a result, the V_OC_, which exhibits a logarithmic dependence on J_0_, diminishes with increasing temperature. V_OC_ decreases from 1.2563 V to 1.003 V in devices with the HTL and from 1.1364 V to 0.7955 V in HTL-free systems when temperature rises from 280 K to 460 K. Besides diminishing V_OC_, heat agitation negatively impacts the FF. Increased temperatures enhance phonon–carrier interactions, resulting in diminished carrier mobility and heightened recombination losses. The effects are exacerbated by a temperature-induced increase in series resistance (R_S_), which impedes efficient charge extraction. The FF significantly declines from 89.45% to 82.44% with HTL and from 88.78% to 80.29% without it. The rise in R_S_ diminishes the effective carrier diffusion length and exacerbates ohmic losses, a phenomenon extensively documented in temperature-dependent photovoltaic research^73,74^. The simultaneous decline in V_OC_ and FF directly results in a decrease in PCE as temperature rises, highlighting the device’s thermal sensitivity. While the J_SC_ may exhibit slight improvement at higher temperatures due to bandgap narrowing and increased carrier production, this enhancement is inadequate to offset the predominant losses resulting from heightened recombination and resistive effects^75^. The observed findings indicate that thermal agitation significantly influences the temperature-dependent performance and stability of Na_2_AuGaBr_6_-based solar cells. These findings underscore the significance of thermal management, interface optimization, and low-resistance contact engineering for ensuring stable device performance at extreme temperatures. The relatively moderate degradation noted in the HTL-assisted configuration highlights the importance of selecting an appropriate transport layer to reduce thermally induced performance losses, thus reinforcing the practical feasibility of the proposed device architecture for real-world photovoltaic applications.

#### Overview of J-V and QE metrics for evaluating PV device performance

**Fig. 14:**
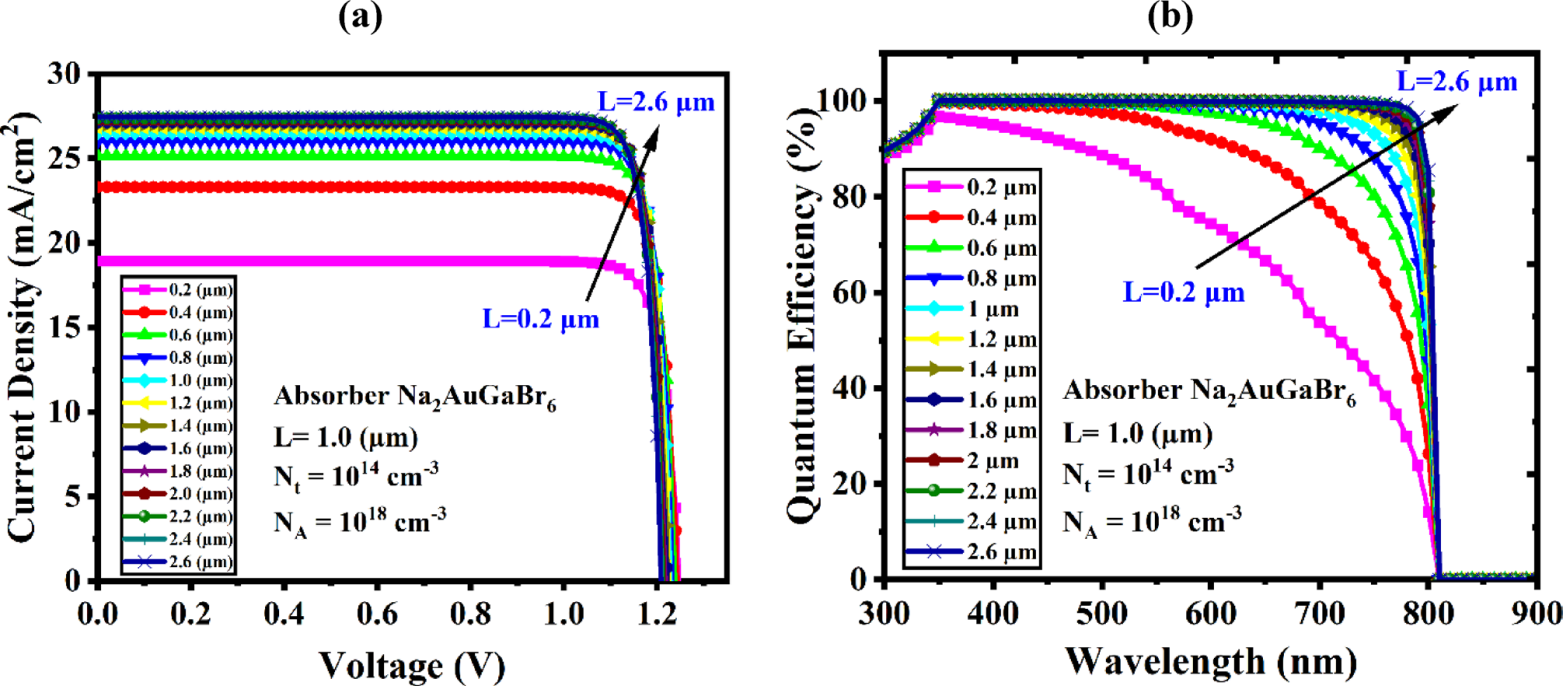
Performance analysis: (**a**) J-V and (**b**) QE profiles for the optimized configuration.

Due to its influence on QE and J-V properties, Na_2_AuGaBr_6_ exhibits significant potential as an absorber layer in SCs^76^. The Na_2_AuGaBr_6_ layer usually absorbs more light when it is thicker, which increases photocurrent generation and, in turn, the J_SC_. Increased recombination losses, which often lower the FF and V_OC_, counteract this advantage and diminish device efficiency overall. According to recent research, the ideal thickness of the Na_2_AuGaBr_6_ absorber is dependent on both external and intrinsic parameters, including fabrication methods, doping concentrations, and device architecture design^77^. By reducing recombination and promoting more efficient charge carrier collection, using a thinner absorber layer can occasionally produce superior outcomes. QE is directly impacted by the absorber depth, which is essential for both photon absorption and photocurrent generation^78^. While increased light absorption in thicker Na_2_AuGaBr_6_ layers tends to boost QE at longer wavelengths, excessive thickness can decrease carrier collecting efficiency, which ultimately results in a fall in QE. To balance these conflicting effects, the absorber thickness must be carefully adjusted. The J-V and QE properties of the Al/FTO/WS_2_/Na_2_AuGaBr_6_/V_2_O_5_/Ni cell structure at different absorber thicknesses are shown in Fig. 14a,b. A PCE of 28.96% was attained in the optimal arrangement, where WS_2_ acts as ETL and V_2_O_5_ as HTL. The current density reaches zero at a forward voltage of 1.235 V, according to the J-V curve in Fig. 14a. This is in line with typical PV behavior, which shows that current declines as voltage increases. The QE profile across a wavelength range of 300–900 nm is shown in Fig. 14b. QE for this device begins at 100% for shorter wavelengths and progressively drops to 0% as the wavelength gets closer to 810 nm. This decrease in QE confirms the direct relationship between photon absorption and current generation efficiency, as does the decrease in photocurrent observed in the J-V characteristics. The observed performance patterns provide more evidence that optimizing absorber thickness is necessary for Na_2_AuGaBr_6_-based PSC to maximize efficiency.

#### Comparative analysis of proposed structure with previous studies

This work compares the performance of the Al/FTO/WS_2_/Na_2_AuGaBr_6_/V_2_O_5_/Ni architecture with previously reported combinations, emphasizing its efficiency and optimization procedure. Configurations utilizing analogous double perovskites, including Cs_2_AgBiBr_6_, Rb_2_LiGaI_6_, Na_2_InAgCl_6_, and Cs_2_AuBiCl_6_, have demonstrated PCEs between 21 and 27%^79–82^, attained through meticulous optimisation of critical parameters such as layer thickness, doping concentration, and defect density. The Al/FTO/WS_2_/Na_2_AuGaBr_6_/V_2_O_5_/Ni device showed significant performance improvement after structural and electrical optimisation, achieving a peak PCE of 28.96%. This arrangement utilizes WS_2_ as ETL and Na_2_AuGaBr_6_ as a lead-free absorber, making it both efficient and environmentally safer than lead-based devices. Table 5 compares the output parameters of the suggested devices with those found in earlier research for comparative comparison. The Al/FTO/WS_2_/Na_2_AuGaBr_6_/V_2_O_5_/Ni architecture either surpasses or equals the performance of prior configurations, presenting a potential, eco-friendly alternative in SC design.

**Table 5:** Review and comparison of theoretical (T) and experimental (E) results in prior SC studies.

Structure	Types of work	PCE (%)	V_OC_ (V)	J_SC_ (mA/cm^2^)	FF (%)	Ref.
TiO_2_/CChl/Cs_2_AgBiBr_6_/spiro-OMeTAD/Au	E	3.11	1.04	4.09	73	^83^
FTO/TiO_2_/Cs_2_AgBiBr_6_/Spiro-OMeTAD/MoO_3_/Ag	E	2.51	1.01	3.82	65	^84^
FTO/TiO_2_/Sb_2_Se_3_/Au	E	2.26	0.52	10.3	42.3	^85^
ITO/CdS/Sb_2_Se_3_/Au	E	6.50	0.43	25.50	59.30	^86^
CsPbI_3_/Cs_0.25_FA_0.75_PbI_3_	E	17.39	1.20	18.91	76	^87^
ITO/TiO_2_/CsPbI_3_/CBTS	T	17.9	0.997	21.07	85.21	^60^
ITO/LBSO/Cs_2_TiBr_6_/CNTS/Au	T	24.82	1.123	26.63	82.94	^88^
Zn/FASnI_3_/CuSCN	T	25.94	1.085	28.12	84.96	^89^
TiO_2_/Cs_2_PdBr_6_/MoO_3_	T	26.00	1.314	20.093	89.55	^90^
TiO_2_/(FA)_2_BiCuI_6_/CNTS	T	26.09	1.38	22.64	83.33	^91^
FTO/ZnO/MAPbI_3_/FAPbI_3_/CBTS/Au	T	31.86	1.48	34.66	84.47	^92^
ITO/CdS/Ag_2_BeSnSe_4_/Spiro-OMeTAD/Au	T	30.4	0.9565	36.1	87.9	^93^
FTO/IGZO/Cs_2_AgInBr_6_/CBTS/Au	T	26.56	1.16	27.28	83.30	^23^
ZnSe/Cs_2_AgInBr_6_/MASnBr_3_	T	26.64	1.156	27.494	83.79	^38^
ITO/WS_2_/Dy_2_NiMnO_6_/CFTS/Au	T	26.72	0.7412	44.679	80.68	^94^
FTO/AZO/Cs_2_AgBiBr_6_/ZnTe	T	26.89	1.50	21.35	83.87	^95^
TiO_2_/Rb_2_LiGaI_6_/Cu_2_O	T	26.48	0.89	31.87	79	^80^
ITO/PEIE/Cs_2_NaAlI_6_/CuAlO_2_/Au	T	25.98	1.38	21.42	87.27	^21^
ITO/ZnO/Cs_2_AuBiCl_6_/CuSbS_2_	T	21.16	0.68	39.94	77.72	^82^
TiO_2_/Na_2_InAgCl_6_/Spiro-OMeTAD	T	25.78	0.85	44.16	68.36	^81^
Al/FTO/WS_2_/Na_2_AuGaBr_6_/V_2_O_5_/Ni	T	28.96	1.229	26.52	88.79	This work

### Machine learning and deep learning analysis

The ongoing progress in photovoltaic technology necessitates a comprehensive understanding of SC performance under diverse physical, material, and environmental conditions. Conventional modelling techniques, although somewhat effective, frequently fail to accurately represent the intricate, nonlinear interactions present in contemporary solar devices. Recently, machine learning (ML) and deep learning (DL) have become significant tools for data-driven analysis, providing high accuracy and predictive capabilities without the need for explicit physical modelling. These techniques identify concealed patterns in extensive datasets, optimize device parameters, and expedite performance evaluation. This work employs Support Vector Regressor (SVR), Random Forest (RF), and Gradient Boosting (GB) as ML techniques, chosen for their efficacy in modelling non-linear interactions and managing small to medium-sized datasets^96,97^. Their interrendersility and capacity to depict intricate connections among SC characteristics and efficiency render them appropriate for this application. Furthermore, an array of DL models was utilized, encompassing Gated Recurrent Units (GRU), Multi-Layer Perceptron (MLP), Artificial Neural Networks (ANN), Deep Neural Networks (DNN), Long Short-Term Memory (LSTM), Recurrent Neural Networks (RNN), Bidirectional LSTM (BiLSTM), and Bidirectional GRU (BiGRU). These models can identify complex patterns and high-dimensional correlations within the dataset, hence improving the comprehension of how material characteristics affect PCE. The resolution of material increments, whether small or coarse - impacts simulation accuracy and computing performance. Higher resolutions yield intricate insights but require substantial processing resources^98^. Coarser resolutions, conversely, diminish simulation duration and storage requirements, albeit at the expense of less detail. This study carefully assesses the effects of coarser resolutions on model accuracy and efficiency, considering the negligible performance fluctuations with minor parameter adjustments and the computing demands of SCAPS-1D. The findings underscore the trade-offs between computational expense and modelling accuracy in predicting solar performance.

#### Data collection and initial characterization

All simulation data were produced utilizing SCAPS-1D (version 3309), integrating essential physical and process aspects that affect photovoltaic device performance. Data preparation and organizing were executed in Python utilizing Pandas and NumPy, guaranteeing reproducibility, precision, and uniformity throughout the dataset. The dataset consists of 236 samples produced by comprehensive SCAPS-1D simulations. The input features are absorber thickness, bandgap, electron affinity, dielectric permittivity, electron and hole mobility, acceptor and bulk defect densities, series and shunt resistances, interface defect density, operating temperature, and the thicknesses of ETL and HTL. The relevant output variables: PCE, V_OC_, J_SC_, and FF were documented to thoroughly assess device performance. A comprehensive statistical description of these parameters, as shown in Table 6, emphasizes their mean values, standard deviations, and ranges of variation, indicating a suitably extensive and well-distributed dataset. In addition to feature preprocessing and redundancy removal, the entire dataset was utilized for training and evaluating ML and DL models. Due to significant physics-based correlations among the variables and the implementation of rigorous cross-validation techniques, this dataset size is sufficient for understanding the predominant nonlinear relationships that dictate device behavior.

**Table 6:** Descriptive statistics of the dataset used for ML and DL model.

Parameters	Count	Mean	Std.	Min.	Max.
Absorber thickness	236	1.5714	0.2	0.2	3.0
Bandgap	236	1.5	0.05	1.3	1.7
Electron affinity	236	3.9	0.05	3.7	4.1
Dielectric permittivity	236	30	2.0	20	40
Electron mobility	236	80	10	10	150
Hole mobility	236	80	10	10	150
Absorber acceptor density	236	7.94 × 10^18^	-	1.0 × 10^10^	1.0 × 10^20^
Absorber defect density	236	8.77 × 10^17^	-	1.0 × 10^10^	1.0 × 10^18^
Series resistance	236	5	0.5	0	10
Shunt resistance	236	8.77 × 10^08^	-	10	1.0 × 10^10^
Interface defect density	236	8.77 × 10^15^	-	1.0 × 10^08^	1.0 × 10^17^
Temperature	236	400	10	300	500
ETL thickness	236	0.16	0.021	0.02	0.3
HTL thickness	236	0.3019	0.025	0.025	0.6

#### Data processing and validation

- *Overview of Dataset Generation*: The dataset utilized in this work was produced via SCAPS-1D (version 3309) simulations, incorporating a variety of physical and procedural characteristics that affect photovoltaic device performance. The dataset was imported into the Python environment with the NumPy and Pandas modules to enable easy data manipulation and repeatability. An overall of 236 simulated device setups was documented, each defined by 14 input parameters, yielding a comprehensive dataset of 3,304 numerical entries.
- *Data Exploration and Anomaly Detection*: A first exploratory data analysis (EDA) was performed utilizing the head(), info(), and description() methods to assess data completeness, parameter distribution, and feature interrelationships. Outliers, missing data, and non-physical results (e.g., negative film thickness or implausible material attributes) were meticulously identified by statistical profiling and visualization methods. Each anomalous value was examined within the framework of photovoltaic device physics to distinguish authentic extremes from computational artefacts. Records that did not satisfy physical plausibility or completeness criteria were eliminated to preserve data integrity and prevent distorted learning behavior.
- *Data Normalization and Feature Scaling*: Due to the diversity in physical units and numerical ranges of the features, feature normalization was implemented to achieve uniform model performance and to avert the dominance of high-magnitude variables. A StandardScaler transformation was applied solely to the training subset to prevent data leaking, subsequently normalizing the validation and test subsets. Tree-based models exhibit relative insensitivity to scaling; however, normalization is crucial for gradient-based deep learning architectures (ANN, DNN, and LSTM), facilitating accelerated convergence, superior generalization, and enhanced training stability.
- *Post-Processing and Dataset Validation*: After preprocessing, the dataset underwent comprehensive revalidation through dimensional checks, summary statistics, and correlation analysis to ascertain structural consistency. The completed dataset maintained its 236 × 14 structure, devoid of absent or erroneous items, so offering a robust basis for future model training, assessment, and predictive analysis.

#### Model validation strategy

- *Data Partitioning and Hold-Out Validation*: A hold-out validation approach was utilized to guarantee a reliable and impartial assessment of the predicted accuracy of all ML and DL models. The dataset, comprising 236 samples and 14 active features, was randomly divided into 80% training data and 20% test data. The training subset was utilized to refine model parameters, whereas the independent test subset was designated for evaluating model generalization on novel data. An internal validation split of 20% of the training data was incorporated during model training for deep learning architectures to monitor convergence, optimize hyperparameters, and mitigate overfitting. All scaling parameters were exclusively fitted on the training subset prior to their application on the validation and test datasets, hence ensuring a method devoid of data leaking and preserving assessment integrity.
- *Performance Metrics and Evaluation Methods*: The performance of the model was assessed using various error measures, such as R^2^, MAE, MSE, RMSE, and MAPE. To improve interpretability, visual diagnostics like feature importance analysis, predicted-versus-actual plots, error distribution histograms, SHAP analysis, correlation heatmap, and train-test comparison curves were utilized. The graphical assessments provide enhanced understanding of each model’s predicted accuracy, learning patterns, and responsiveness to various input characteristics.
- *Cross-Validation Considerations*: Despite the common preference for k-fold cross-validation due to its statistical robustness, this study opted for an 80:20 split owing to the moderate dataset size and the computing demands of training numerous deep learning models, including LSTM, RNN, and BiLSTM. This method guaranteed an adequate quantity of samples for both training and independent assessment while preserving computational efficiency. The incorporation of internal validation for deep learning models enhanced reliability without substantially elevating computing requirements.
- *Unified Validation Framework and Model Reliability*: This integrated validation approach offered a visible, consistent, and rigorous evaluation of model performance. It guaranteed that the given metrics accurately represent the inherent predictive capacity of the ML and DL models, devoid of distortions caused by overfitting or arbitrary data segmentation. This framework created a solid basis for assessing the generalization capabilities of prediction models and for enhancing the use of ML in optimizing Na_2_AuGaBr_6_-based double PSCs.

#### Model training and selection: a comparative analysis

**Fig. 15:**
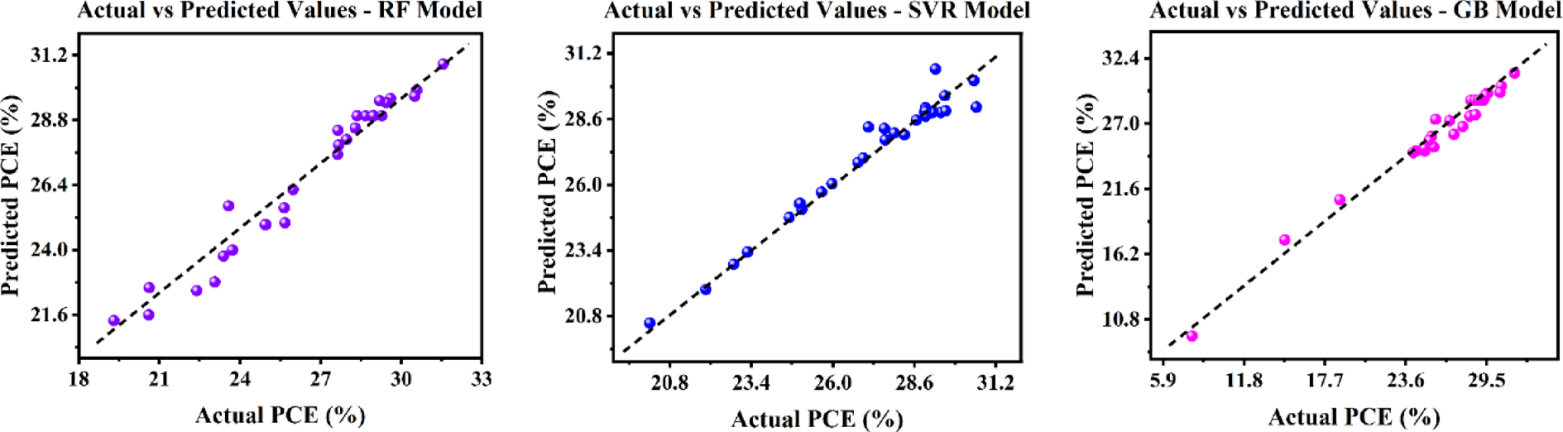
Assessment of ML model predictions via test parity plots.

After data preprocessing, several regression algorithms were implemented and systematically benchmarked. The following section offers a comparative analysis of several DL and ML approaches. The test parity plots for the ML models are depicted on Fig. 15. Additionally, Table 7 consolidates the performance metrics associated with these models. To assess predictive efficacy, various statistical metrics were utilized, including R^2^, MAE, MSE, RMSE, and MAPE. Each indicator offers unique insights into model correctness, forecast precision, and error magnitude.

Optimal model performance is typically indicated by reduced MSE, RMSE, and MAPE values, along with elevated R^2^ scores^99^. According to Table 7 and the parity plots, the GB approach surpassed the other ML models, specifically RF and SVR. GB attained a R^2^ of 0.954, an MSE of 0.756, an MAPE of 0.0218, and a RMSE of 0.869, indicating exceptional predictive performance. On the other hand, of the three models, the SVR technique had the highest error metrics, with an MSE of 1.460, RMSE of 1.208, and MAPE of 0.0266, despite a R^2^ of 0.921, suggesting inadequate generalization despite an apparently sufficient explanation of variation.

**Table 7:** Comparative analysis of the efficacy of multiple algorithms.

Model	R^2^	MAE	MSE	RMSE	MAPE
RG	0.935	0.335	0.539	0.734	0.0242
SVR	0.921	0.675	1.460	1.208	0.0266
GB	0.954	0.425	0.756	0.869	0.0218
GRU	0.890	1.739	1.1617	1.408	0.0723
BiGRU	0.900	1.550	1.1453	1.384	0.0517
LSTM	0.861	1.295	3.908	1.977	0.0532
BiLSTM	0.901	1.884	1.4702	1.834	0.0761
RNN	0.851	1.635	0.5712	1.390	0.0614
ANN	0.929	1.304	0.3131	1.370	0.0499
DNN	0.897	1.527	0.3388	1.841	0.0623
MLP	0.891	2.122	2.4475	1.947	0.0760

**Fig. 16:**
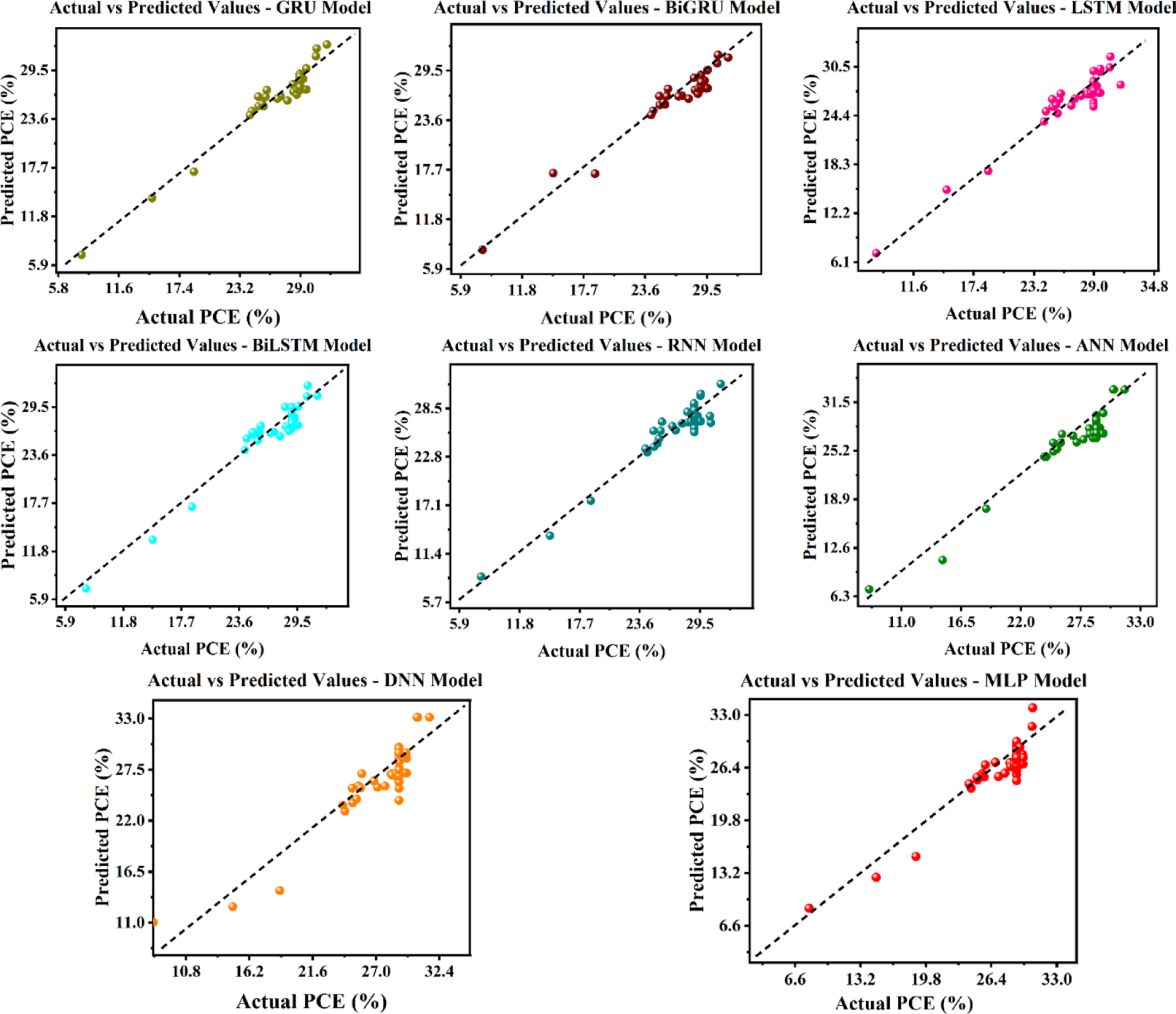
Comparison of actual and predicted outputs for DL models utilizing parity plots.

Subsequent investigation encompassed the training and assessment of eight DL models. Figure 16 depicts the relevant parity plots, with their performance metrics.

The highest accuracy was achieved by the ANN model, recording MAE of 1.304, an MSE of 0.3131, RMSE of 1.370, MAPE of 0.0499, and an excellent R^2^ value of 0.929. Conversely, RNN and LSTM models had the largest error rates and the lowest R^2^ values, 0.851 and 0.861, respectively, signifying inadequate appropriateness for the task. Alternative models, including the simple MLP, DNN, GRU, BiLSTM, and BiGRU, exhibited moderate performance, with MSE values spanning from 0.3388 to 3.908 and R^2^ scores ranging from 0.890 to 0.901. The evaluation findings repeatedly demonstrate that ANN surpasses all other models in minimizing error and maximizing predictive accuracy, hence confirming it as the most appropriate model for outcome prediction.

#### Proposed gradient boosting (GB) model for forecasting SC efficiency

The Gradient Boosting (GB) model demonstrated superior prediction accuracy in estimating the PCE of Na_2_AuGaBr_6_-based DPSCs, surpassing all other ML and DL models evaluated in this research. GB was chosen as an optimal prediction framework because of its shown capacity to identify intricate, non-linear relationships among material properties, device architectures, and PV performance indicators. As an ensemble learning algorithm, GB incrementally builds weak learners, usually shallow decision trees where each subsequent model is trained to reduce the residual errors of its predecessors using gradient descent optimization on a specified loss function. This iterative refining method allows GB to generalize effectively across high-dimensional datasets while preserving resilience against overfitting. Before training, input variables were standardized with a StandardScaler to guarantee numerical stability and expedite convergence. This configuration offered an optimal equilibrium between model intricacy and prediction generalization. In comparison to other algorithms, the GB model consistently produced superior outcomes. In contrast to deep models, which frequently function as “black boxes” requiring significant hyperparameter optimization, Gradient Boosting provides interpretability via explicit feature importance metrics, enabling researchers to discern and prioritize key elements affecting PCE. Furthermore, GB operates efficiently with moderate dataset sizes, rendering it especially beneficial for computational or experimental PV research when extensive datasets are not easily accessible. Table 8 summarizes that the PCE derived from SCAPS-1D was 28.96%, whereas the GB model forecasted 28.1874% utilizing the comprehensive feature set and 27.6681% when trained solely on five critical parameters including absorber thickness, acceptor density, defect density, interface defect density, and temperature exhibiting remarkable consistency and robustness.

**Table 8:** ML-predicted vs. actual PCE (selected inputs: thickness, acceptor density, defect density, interface defect, and temperature) (A = Actual, P = Predicted).

Feature vector	V_OC_		J_SC_		FF		PCE	
	A	P	A	P	A	P	A	P
All eighteen features according to Table 2	1.229	1.2054	26.52	26.5215	88.79	88.1730	28.96	28.1874
(1.0, 1 × 10^18^, 1 × 10^14^, 1 × 10^12^, 300) (only 5 features)	1.229	1.1878	26.52	26.5196	88.79	87.6623	28.96	27.6681

These findings validate the GB model’s capacity to accurately interpolate device performance while preserving interpretability. The proposed GB strategy creates a dependable, efficient, and transparent prediction framework for enhancing PSC performance and directing data-driven material and device developments towards sustainable, lead-free photovoltaic systems.

#### Evaluation of diagnostic and interpretability aspects of the proposed model

##### Quantile-Quantile (Q-Q) plot analysis

**Fig. 17:**
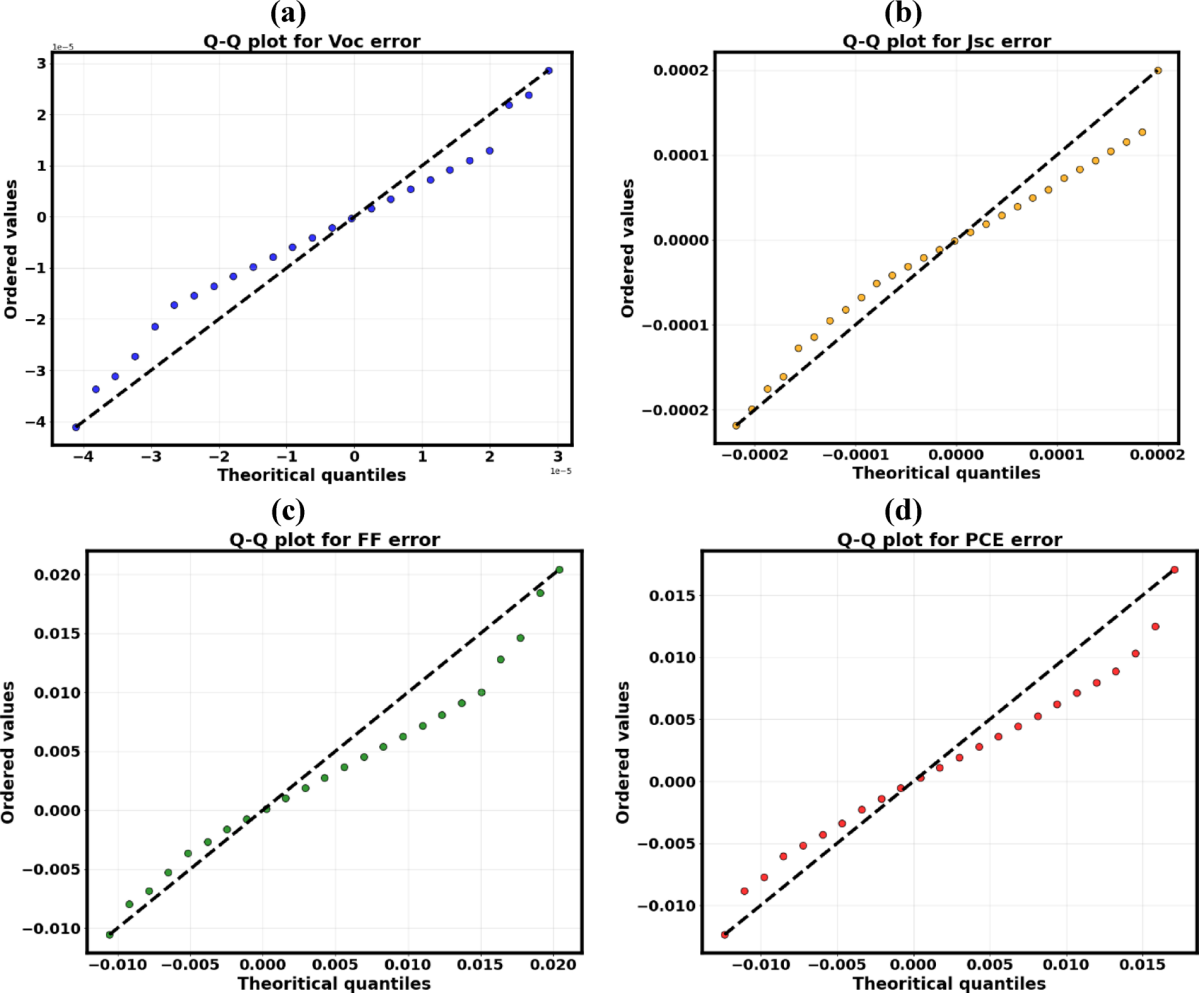
Q–Q plots showing the normality of prediction errors from the GB model for (**a**) V_OC_, (**b**) J_SC_, (**c**) FF, and (**d**) PCE.

A comprehensive validation system guaranteed the statistical integrity and efficacy of the GB regression model. Q-Q diagrams were created for all four PV output parameters to evaluate the normality of residuals and confirm the statistical assumptions of regression modeling, as seen in Fig. 17a–d. The data points displayed an almost perfect linear correlation along the 45° reference line, validating that the model residuals roughly conform to a normal distribution. This tendency indicates that the discrepancies between predicted and actual values are predominantly random rather than systematic, suggesting a lack of skewness or kurtosis in the error structure. The standardized distribution of residuals supports a fundamental assumption of regression analysis, demonstrating that random errors outweigh variations and confirming the lack of structural bias or heteroscedasticity in the model. The Q-Q plot and residual analysis collectively confirm the statistical reliability, diagnostic robustness, and generalization stability of the proposed GB model. The existence of symmetrical error distribution and minimum residual variance further emphasizes the model’s capacity to accurately recreate complicated, nonlinear photovoltaic reactions. The diagnostic findings robustly affirm the methodological integrity of the GB workflow, underscoring its appropriateness for high-throughput, data-driven prediction and optimization of lead-free PSCs.

##### Distribution of prediction errors analysis

**Fig. 18:**
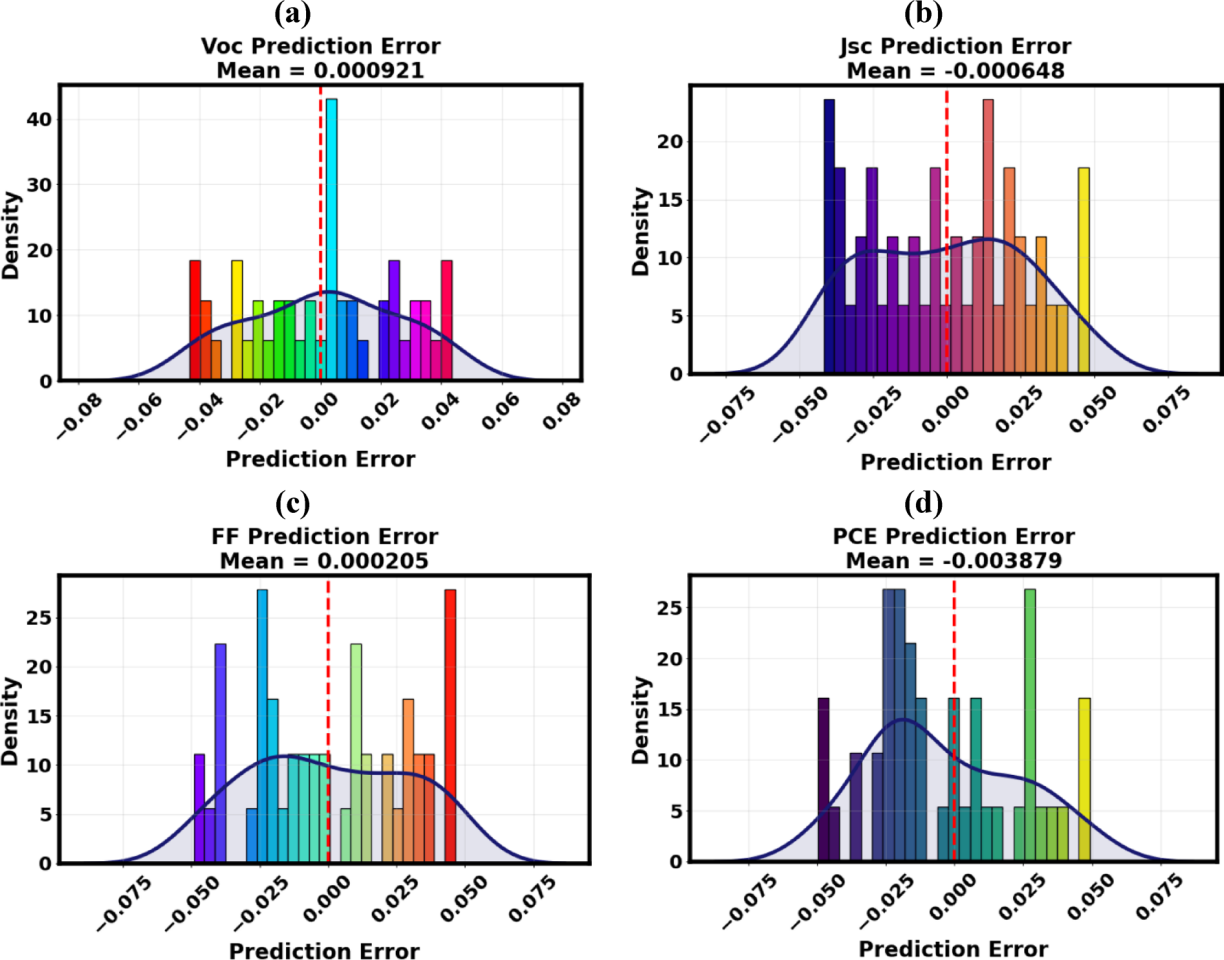
Distribution of prediction errors from the GB model for (**a**) V_OC_, (**b**) J_SC_, (**c**) FF, and (**d**) PCE.

The distribution of prediction errors was analyzed to assess the model’s calibration accuracy, predictive reliability, and internal consistency. Figure 18a–d depicts histograms that represent the density distributions of residuals between ML-predicted values and simulated values for all four principal performance metrics. In these graphs, the x-axis signifies the prediction error (i.e., the disparity between expected and simulated outcomes), whilst the y-axis indicates the estimated frequency of incidence. The red dashed lines represent the average error levels for each statistic. The distributions display sharply centered, narrow peaks around zero, with minimal mean deviations (e.g., mean error for V_OC_ = 0.000921 and for PCE = − 0.003879), signifying that prediction errors are statistically unbiased and symmetrically distributed. The near-zero centering verifies the lack of systematic overestimation or underestimation, while the narrow dispersion indicates little random variability and robust model calibration.

The balanced, symmetric error pattern indicates that the GB regressor achieves a balance between overpredictions and underpredictions, hence maintaining consistent performance across unfamiliar setups. Among the assessed models, the GB algorithm exhibited exceptional error management, evidenced by closely grouped residuals across all PV criteria. These diagnostic results affirm the GB framework as an effective and transparent predictive instrument for expediting the design, optimization, and performance prediction of next-generation perovskite and hybrid solar cell technologies.

#### Feature importance and physical corroboration

With robust confidence established from the model’s exceptional cross-validation results in the preceding section, the GB model was subsequently utilized to identify the most significant factors affecting solar cell efficiency. To accomplish this, two complimentary analytical approaches were employed: the inherent Feature Importance metric of the GB algorithm and the more comprehensive Shapley additive explanations (SHAP) analysis. The feature significance evaluations directly quantify each variable’s contribution to the predictive model, whereas SHAP values provide a more elaborate, instance-specific interpretation of how each input feature affects the model’s output^100,101^. Together, these methodologies facilitated a comprehensive and interpretable evaluation of the material and device factors that most significantly influenced the predicted PCE.

##### Evaluation of feature relative importance in model performance

**Fig. 19:**
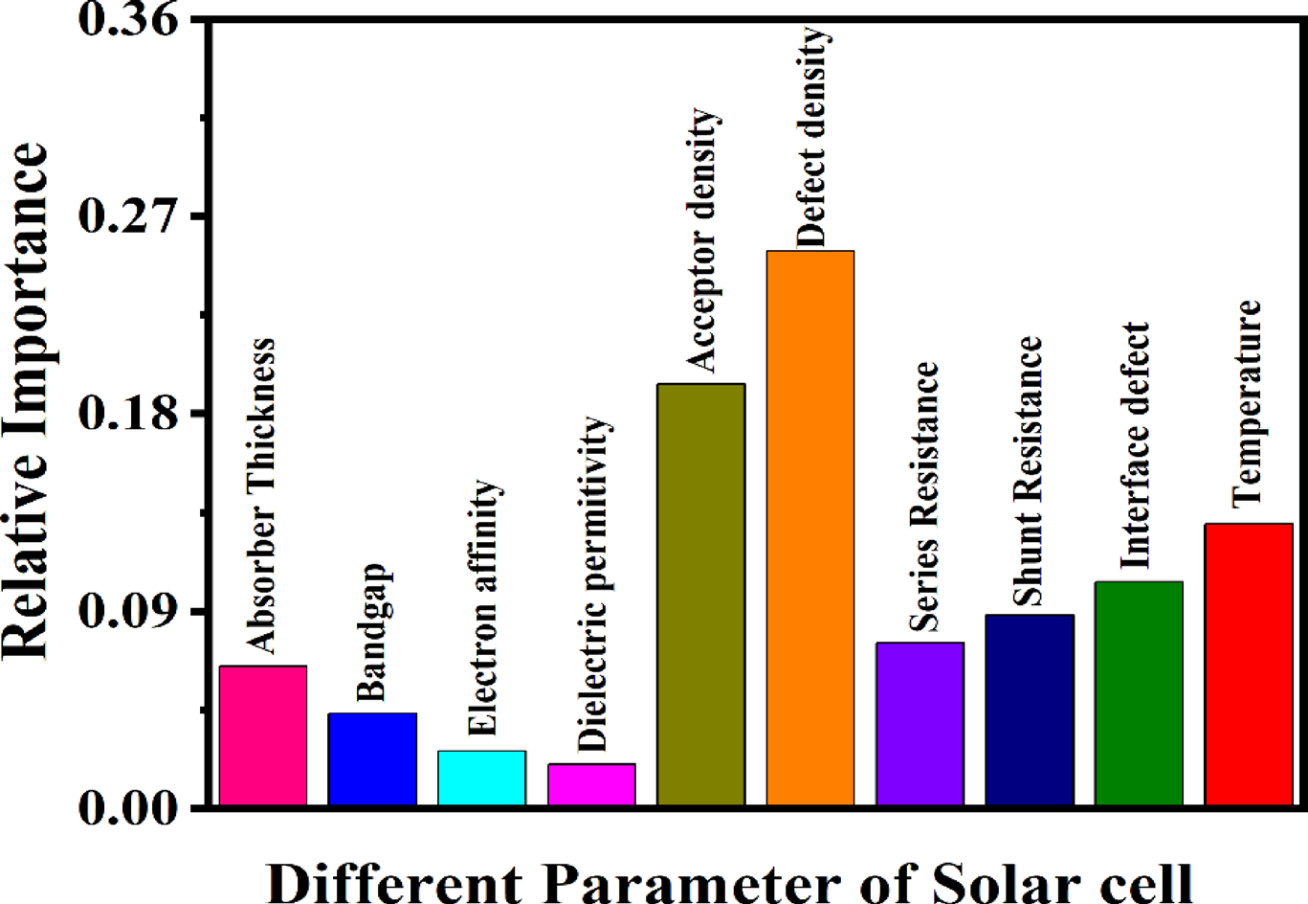
Feature significance for Al/FTO/WS_2_/Na_2_AuGaBr_6_/V_2_O_5_/Ni cells in GB models: Bar chart visualization.

Feature selection is essential to identifying the most critical parameters affecting SC performance, hence enhancing the model’s predicted accuracy. In the GB model, the relative relevance of features is determined by their contribution to the reduction in the loss function throughout the boosting phase. The significance of a feature is assessed by its efficacy in diminishing residual errors (i.e., impurity) during target variable prediction. The reduction is quantified by the average decline in the loss function (e.g., MSE) at each split in the decision trees throughout model training. Figure 19 demonstrate the feature importance of proposed soler cell structure. The GB model identifies critical factors such as defect and acceptor density, interface features, and temperature, which substantially influence the performance of high-efficiency DPSCs.

Other crucial variables include layer thickness, bandgap, shunt-series resistance, dielectric permittivity, and electron affinity. These attributes enhance the model’s comprehension of the non-linear correlations between input variables and the anticipated PCE. The identified feature importances are essential for influencing material optimisation and experimental design to improve solar cell performance. The feature-important values of the GB model offer valuable insights into the material properties that most substantially influence device efficiency.

##### Shapley additive explanations (SHAP) analysis

**Fig. 20:**
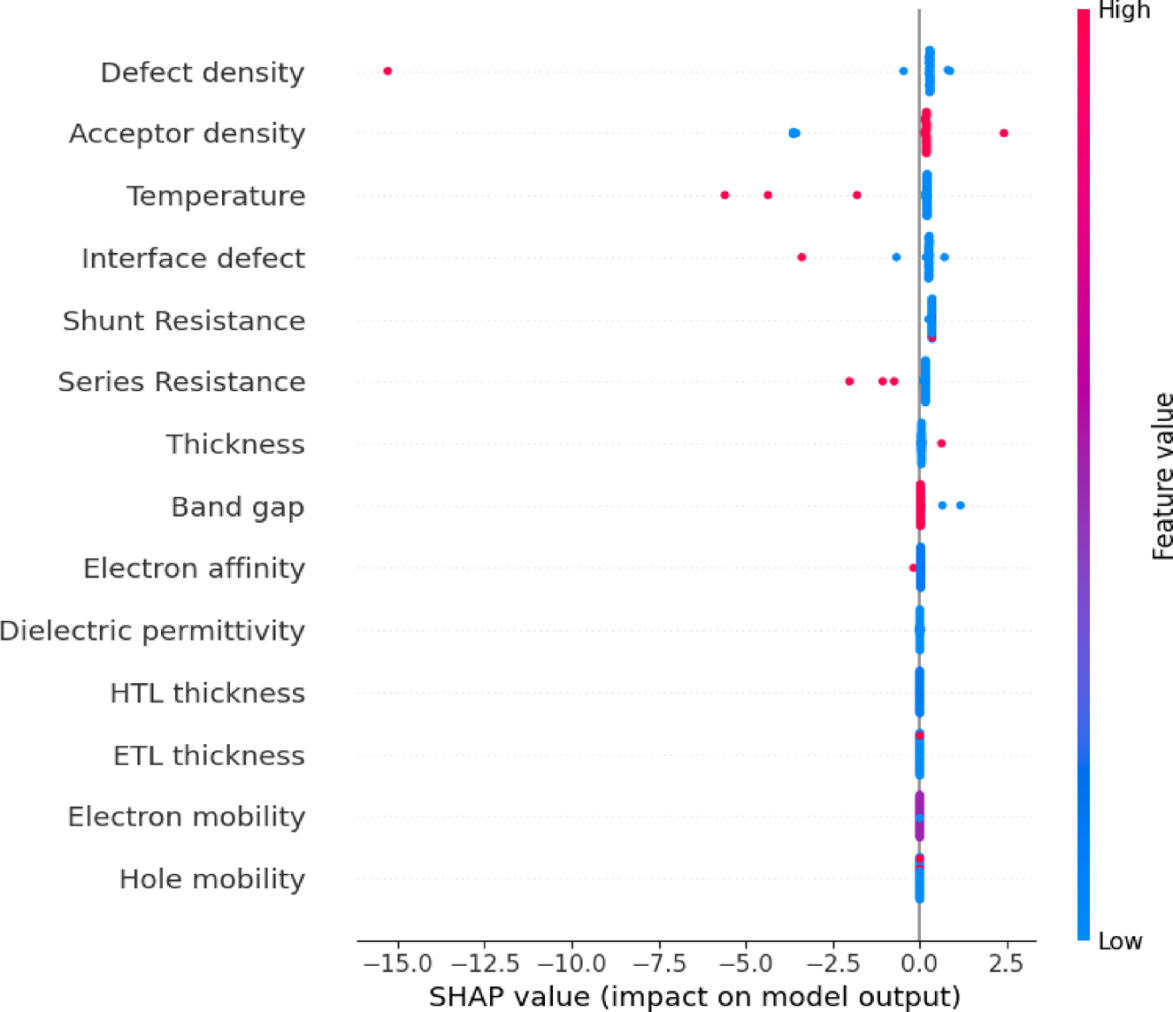
SHAP analysis of GB regressor for predicting device PCE.

A SHAP analysis was performed on the GB model to evaluate the relative significance of features and their influence on the model’s predictions. SHAP is an effective method that quantifies the contribution of each feature to the model’s output, providing insights into the significance and amount of each feature’s impact^102^. Figure 20 demonstrates that the SHAP analysis produces numerous predictions and assesses their contributions using a comparative method, offering an extensive overview of feature significance. Within the framework of the GB model, the SHAP analysis integrates feature significance with the effect of individual features, represented in a summary plot that orders the characteristics by their descending influence on the anticipated PCE.

The analysis indicated that the three most significant features, defect density, acceptor density, and temperature constitute the overall effect on device efficiency. These findings are essential for directing experimental endeavors, enabling researchers to priorities the optimisation of critical parameters to enhance PCE. The integration of SHAP analysis and GB feature importance offers a comprehensive perspective on the model’s decision-making process and a pragmatic guide for optimizing materials and devices.

##### Analysis of correlation heatmap

**Fig. 21:**
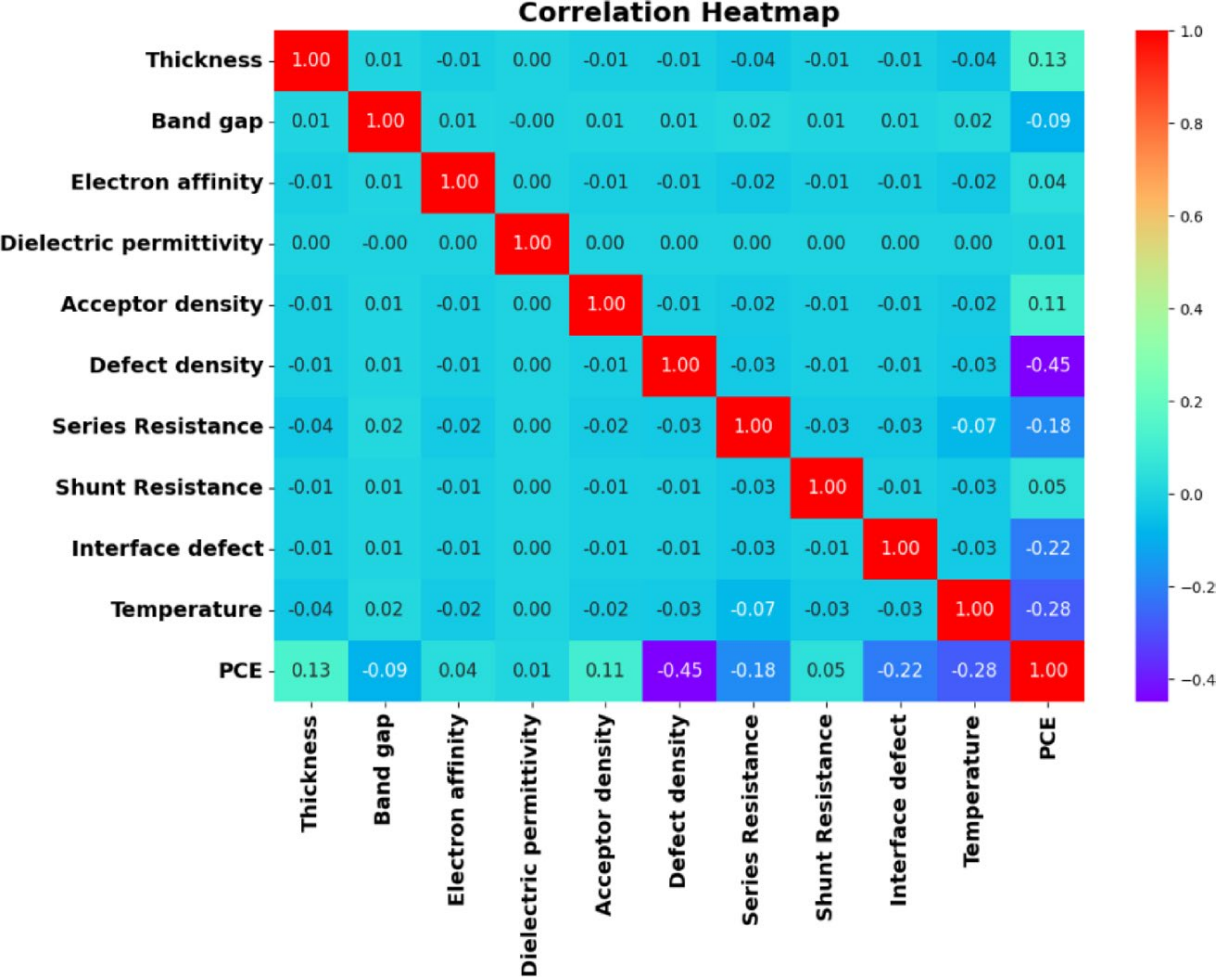
Correlation heatmap of the proposed SC configuration illustrating correlations among several variable factors.

Correlation analysis is a robust statistical method employed to evaluate the magnitude and pattern of correlations among variables. Figure 21 illustrates the correlation heatmap, depicting the correlations between various parameters and the target variable (PCE). The heatmap illustrates both positive and negative correlations, with values spanning from + 1 to -1, but values close to zero signify weak or negligible correlation. The heatmap indicates a significant negative correlation between PCE and defect density (-0.45), implying that an increase in defect density corresponds with a drop in power conversion efficiency, aligning with the expectation that defects impair material performance. A minor negative correlation exists between PCE and series resistance (− 0.18), suggesting that heightened resistive losses negatively impact device efficiency. Elevated series resistance impedes charge carrier mobility through the device layers, resulting in heightened ohmic losses and a diminished FF, which collectively constrain total solar efficiency. Series resistance demonstrates an inverse correlation with carrier-related parameters, as increased carrier densities enhance electrical conductivity and facilitate more effective charge extraction at the interfaces^103^. The improved carrier transport diminishes voltage losses in the device and alleviates resistive effects, thus enhancing PCE. A moderate negative correlation of -0.28 is noted between PCE and temperature, indicating that elevated temperatures may diminish power conversion efficiency. PCE exhibits minor positive correlations with thickness (0.13) and acceptor density (0.11), suggesting that increases in these variables may have a marginally beneficial impact on efficiency. Additional characteristics, such as band gap, electron affinity, dielectric permittivity, and shunt resistance, exhibit weak or negligible correlations with PCE, indicating that these factors have minimal influence on the target variable within this dataset. The correlation heatmap offers significant insights into the correlations between input features and power conversion efficiency, aiding in feature selection and guiding future model development for enhanced SC design.

#### Model performance evaluation

**Fig. 22:**
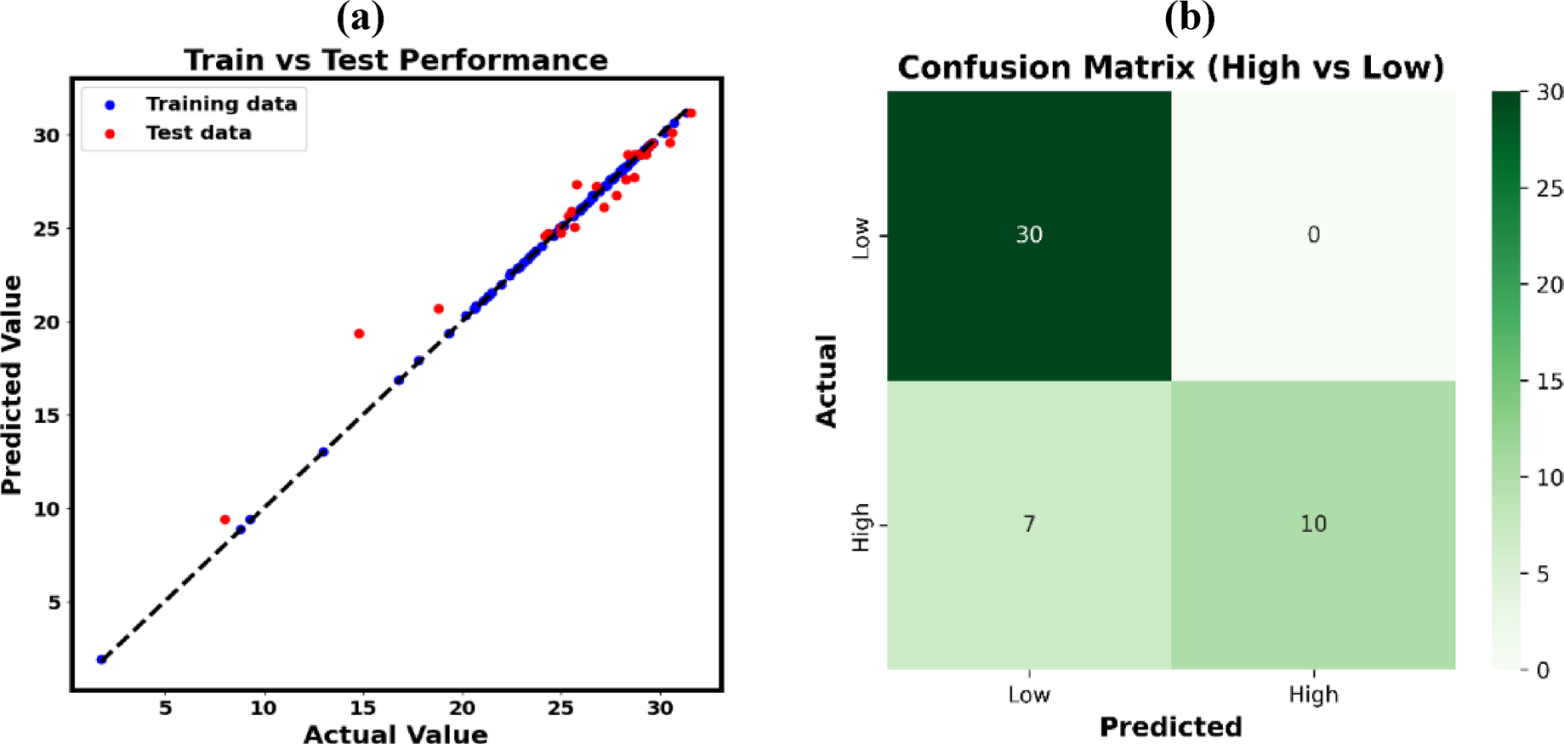
The performance of proposed model analyzed through (**a**) parity plot and (**b**) confusion matrix for suggested SC structure.

The performance of the GB model was assessed by the train versus test performance plot and the confusion matrix, offering complimentary insights into its predictive efficacy. Figure 22a presents the training versus testing plot, both training and test data points are closely grouped around the best prediction line, indicating that the model effectively reflects the underlying data patterns. The proximity of training and test locations indicates limited overfitting and robust generalization capability. Minor discrepancies in the test predictions indicate areas for refinement to attain closer congruence with actual values. The confusion matrix presented in Fig. 22b provides a quantitative assessment of the classification performance across different categories. The model attains flawless classification for the “Low” class, exhibiting zero false positives and a substantial number of true positives, hence demonstrating exceptional dependability in identifying low outcomes. Conversely, the “High” class demonstrates misclassification mistakes, notably 7 false negatives, when true high values were incorrectly categorized as low. Although true positives prevail, these misclassifications diminish recall for the “High” category, suggesting the model’s comparative inadequacy in identifying all instances of high values. Collectively, these results demonstrate a solid predictive framework: the train versus test comparison validates great generalization, while the confusion matrix highlights specific deficiencies in recall for the “High” class. Modifying thresholds or correcting imbalances could substantially decrease false negatives, hence enhancing the model’s robustness and practical applicability in supply chain applications.

## Feasibility of fabrication and practical implementation of the Al/FTO/WS_2_/Na_2_AuGaBr_6_/V_2_O_5_/Ni solar cell

To validate the computationally optimized photovoltaic performance of the Al/FTO/WS_2_/Na_2_AuGaBr_6_/V_2_O_5_/Ni device architecture, a comprehensive experimental fabrication and characterization roadmap is proposed. This framework combines material synthesis, device assembly, interface engineering, optical optimization, and stability assessment, thereby creating a viable pathway for converting simulation and machine learning predictions into practical, high-efficiency, lead-free PSCs.

### Synthesis and fabrication of Na_2_AuGaBr_6_ absorber material

The Na_2_AuGaBr_6_ double perovskite absorber can be manufactured using solution-based processing, solid-state reaction, or vapor-assisted deposition processes, contingent upon the required film quality and scalability. Solution processing is especially appealing because of its compliance with low temperatures, cost efficiency, and appropriateness for large-area production. Exact regulation of precursor stoichiometry, solvent choice, and crystallization kinetics is crucial to guarantee phase purity and inhibit the emergence of secondary phases.

Regulated annealing in inert or halide-rich environments can augment crystallinity and facilitate defect passivation, aligning with the low defect densities posited in the SCAPS-1D simulations. X-ray diffraction (XRD) will verify the cubic phase of Na_2_AuGaBr_6_, while scanning electron microscopy (SEM) and atomic force microscopy (AFM) will elucidate grain size, surface roughness, and film continuity. Elemental uniformity and chemical composition can be assessed by energy-dispersive X-ray spectroscopy (EDS) and X-ray photoelectron spectroscopy (XPS).

### Device architecture and layered fabrication

The suggested solar cell utilizes a planar heterojunction structure of Al/FTO/WS_2_/Na_2_AuGaBr_6_/V_2_O_5_/Ni, manufactured within an inert glovebox environment to avert deterioration from oxygen and moisture.

#### Preparation of substrate and front electrode

Fluorine-doped tin oxide (FTO) coated glass substrates undergo a progressive cleaning process involving detergent, acetone, isopropanol, and deionized water, succeeded by oxygen plasma treatment to enhance surface wettability and eliminate organic impurities. An aluminum front electrode is integrated to guarantee effective current collection and compatibility with transparent conductive oxide coatings.

#### Deposition of WS_2_ ETL

Tungsten disulfide (WS_2_) is chosen as ETL owing to its elevated electron mobility, chemical stability, and advantageous conduction band alignment with Na_2_AuGaBr_6_, as validated by band-offset analysis. WS_2_ films may be deposited via chemical vapor deposition (CVD), radio-frequency sputtering, or solution-phase exfoliation methods to get dense, pinhole-free layers. The ETL thickness is adjusted to achieve efficient electron extraction while minimizing parasitic optical absorption.

#### Deposition of Na_2_AuGaBr_6_ absorber layer

The Na_2_AuGaBr_6_ absorber layer is applied on the WS_2_ ETL by spin coating or vapor-assisted deposition, thereafter, undergoing controlled thermal annealing to improve crystallinity and promote grain development. Thickness optimization, informed by SCAPS-1D simulations, guarantees adequate light absorption while reducing bulk recombination and transport losses.

#### Deposition of V_2_O_5_ HTL

Vanadium pentoxide (V_2_O_5_) is utilized as HTL due to its elevated work function, superior hole selectivity, and chemical compatibility with Na_2_AuGaBr_6_. Thin V_2_O_5_ layers can be deposited through thermal evaporation or sputtering, creating a uniform interface that enhances efficient hole extraction and mitigates interfacial recombination.

#### Formation of back contacts

Nickel (Ni) is placed as the rear electrode by thermal evaporation or sputtering methods. Ni establishes a robust, low resistance ohmic contact with V_2_O_5_, facilitating efficient hole collection and maintaining long-term operational stability.

### Interface engineering and defect passivation

The quality of the interface is a crucial factor influencing device efficiency and stability. The WS_2_/Na_2_AuGaBr_6_ and Na_2_AuGaBr_6_/V_2_O_5_ interfaces can be enhanced via surface passivation techniques, including moderate plasma treatment, self-assembled monolayers, or ultrathin insulating interlayers. These methods diminish interfacial defect concentrations, inhibit non-radiative recombination, and facilitate the elevated fill factors anticipated by the device models.

Advanced interfacial characterization techniques, such as Kelvin probe force microscopy (KPFM) and conductive atomic force microscopy (c-AFM), can be utilized to elucidate local potential distribution and charge transport channels at the nanoscale.

### Optical engineering and anti-reflective coatings

To optimize real-device performance and sustain efficiency under practical operating situations, optical losses must be reduced. The application of anti-reflective coatings (ARCs), such as MgF_2_, Al_2_O_3_, HfO_2_, ZnS, or SiO_2_, on the glass surface is suggested to diminish Fresnel reflection and enhance photon coupling into the absorber layer^104^. Furthermore, textured FTO substrates or graded refractive index coatings may be investigated to enhance light trapping and prolong optical path lengths within the Na_2_AuGaBr_6_ absorber.

### Electrical and photovoltaic characterization

The photovoltaic performance will be assessed under typical AM 1.5G lighting, producing essential metrics such as PCE, V_OC_, J_SC_, and FF. External quantum efficiency (EQE) measurements will evaluate the wavelength-dependent efficacy of carrier collecting.

Electrical characterization methods, including impedance spectroscopy, capacitance–voltage (C–V) profiling, and time-resolved photoluminescence (TRPL), will elucidate charge transport resistance, recombination kinetics, carrier lifetime, and defect-related losses.

### Evaluation of stability and reliability testing

Thorough stability testing is crucial to assess device resilience in practical situations. Environmental stability will be evaluated via regulated humidity and oxygen exposure, whereas thermal stability assessments will incorporate increased temperature cycling. Operational stability will be assessed under continuous illumination utilizing maximum power point tracking (MPPT) to evaluate performance deterioration over time. Encapsulation techniques and moisture barriers can be utilized to improve long-term stability.

### Scalability and market potential

The compatibility of Na_2_AuGaBr_6_ and transport layers with low-temperature, solution-processable methods like blade coating and slot-die coating underscores the potential for scale production. The Al/FTO/WS_2_/Na_2_AuGaBr_6_/V_2_O_5_/Ni design, incorporating a lead-free composition, materials abundant in the earth, and optical enhancement methods, offers a viable avenue for environmentally sustainable, high-efficiency solar systems.

## Conclusion

This research utilized DFT calculations and SCAPS-1D simulations to evaluate the efficacy of the Al/FTO/ETL/Na_2_AuGaBr_6_/HTL/Ni multilayer solar cell configuration. A comprehensive collection of HTLs and ETLs was examined across 48 device architectures to ascertain the best configuration. The findings indicated that V_2_O_5_ as HTL, in conjunction with WS₂ as ETL, exhibited enhanced performance relative to other combinations examined. Multiple device characteristics, including layer metal contact, thickness and doping concentration, were optimized to enhance performance. The Na_2_AuGaBr_6_ absorber layer was configured with a thickness of 1000 nm and a doping concentration of 10^18^ cm^− 3^, whilst the V_2_O_5_ HTL measured 100 nm in thickness, and the WS_2_ ETL was 50 nm thick. The calculations reveal that the defect densities at the Na_2_AuGaBr_6_ layer and interfaces (V_2_O_5_/Na_2_AuGaBr_6_ and Na_2_AuGaBr_6_/WS_2_) were 10^15^ cm^− 2^ and 10^12^ cm^− 2^, respectively. The optimized Al/FTO/WS_2_/Na_2_AuGaBr_6_/V_2_O_5_/Ni configuration attained a PCE of 28.96%, with a V_OC_ of 1.2298 V, J_SC_ of 26.5209 mA/cm^2^, and FF of 88.79%. These findings indicate that this design may be employed for high-efficiency SCs in the PV sector. To ascertain the most precise predictive model for SC devices, eight DL and three ML algorithms were assessed in total. The GB model exhibited exceptional predictive ability, attaining a remarkable R^2^ of 0.954 and a low MSE of 0.756. The interpretability methods underscored the promise of Na_2_AuGaBr_6_ an effective light-absorbing layer in PSCs. Future endeavors should concentrate on optimizing material quality, minimizing recombination losses, regulating doping levels to boost carrier mobility, and modifying layer thickness to improve light absorption and conveyance. Furthermore, the application of anti-reflection coatings will enhance overall efficiency.

## Supplementary Information

Below is the link to the electronic supplementary material.

Supplementary Material 1

## Data Availability

The data that supports the findings of this study are available within the article and its Supporting data set and can be accessed at: https://tinyurl.com/32jw8v7w.
