# Supplementary material for: Investigating the optoelectronic properties and photovoltaic performance of Na2AuGaBr6 based double perovskite solar cells via numerical simulation and AI techniques
Source: Sci Rep. 2026 Feb 27;16:11218. doi: 10.1038/s41598-026-41519-x (PMC13046740; doi:10.1038/s41598-026-41519-x)
Supplement: Supplementary file 1 — Supplementary Material 1 [file 41598_2026_41519_MOESM1_ESM.docx]

**Supporting Information**

**Investigating the Optoelectronic Properties and Photovoltaic Performance of Na_2_AuGaBr_6_ Based Double Perovskite Solar Cells via Numerical Simulation and AI Techniques**

Bipul Chandra Biswas^1^, Asadul Islam Shimul^1^, Indrojit Paul^1^, S. AlFaify^2^, Mohamed Benghanem^3^, Md. Azizur Rahman^4^, Gideon F. B. Solre^5*^, Noureddine Elboughdiri^6^

*^1^Department of Electrical and Electronic Engineering,* *Gopalganj Science and Technology University, Gopalganj-8105, Bangladesh.*

*^2^Department of Physics, College of Sciences, King Khalid University, P.O. Box 960, AlQura’a, Abha, Saudi Arabia.*

*^3^Physics Department, Faculty of Science, Islamic University of Madinah, Madinah 42351, Saudi Arabia.*

*^4^Department of Electrical and Electronic Engineering, Begum Rokeya University, Rangpur 5400, Bangladesh.*

*^5^Department of Chemistry, Thomas J. R. Faulkner College of Science, Technology, Environment and Climate Change, University of Liberia, 00231 Monrovia, Montserrado County, Liberia.*

*^6^Chemical Engineering Department, College of Engineering, University of Ha’il, P.O. Box 2440, Ha'il 81441, Saudi Arabia.*

Correspondence: *g.f.b.solre@gmail.com, gideonfbsolre@gmail.com (Gideon F. B. Solre)

**Table S1.** The simulated photovoltaic performance results for Na_2_AuGaBr_6_-based device structures incorporating six electron transport layers (ETLs) and eight-hole transport layers (HTLs).

| **SL. No** | **Device Structure** | **V_OC_ (V)** | **J_SC_**  **(mA/cm^2^)** | **FF (%)** | **PCE (%)** |
| --- | --- | --- | --- | --- | --- |
| 1 | Al/FTO/TiO_2_/Na_2_AuGaBr_6_/CFTS/Ni | 1.117 | 24.73 | 88.22 | 24.39 |
| 2 | Al/FTO/TiO_2_/Na_2_AuGaBr_6_/CuI/Ni | 1.146 | 25.99 | 88.45 | 26.37 |
| 3 | Al/FTO/TiO_2_/Na_2_AuGaBr_6_/NiO/Ni | 1.181 | 26.43 | 87.76 | 27.4 |
| 4 | Al/FTO/TiO_2_/Na_2_AuGaBr_6_/CuSbS_2_/Ni | 1.096 | 22.59 | 87.49 | 21.67 |
| 5 | Al/FTO/TiO_2_/Na_2_AuGaBr_6_/V_2_O_5_/Ni | 1.202 | 26.51 | 86.83 | 27.69 |
| 6 | Al/FTO/TiO_2_/Na_2_AuGaBr_6_/MoTe_2_/Ni | 1.156 | 26.19 | 88.36 | 26.76 |
| 7 | Al/FTO/TiO_2_/Na_2_AuGaBr_6_/Sb_2_S_3_/Ni | 1.173 | 26.4 | 88.01 | 27.27 |
| 8 | Al/FTO/TiO_2_/Na_2_AuGaBr_6_/CuO/Ni | 1.106 | 23.82 | 87.96 | 23.19 |
| 9 | Al/FTO/ZnO/Na_2_AuGaBr_6_/CFTS/Ni | 1.116 | 24.73 | 88.07 | 24.32 |
| 10 | Al/FTO/ZnO/Na_2_AuGaBr_6_/CuI/Ni | 1.144 | 25.99 | 88.15 | 26.23 |
| 11 | Al/FTO/ZnO/Na_2_AuGaBr_6_/NiO/Ni | 1.175 | 26.43 | 87.33 | 27.15 |
| 12 | Al/FTO/ZnO/Na_2_AuGaBr_6_/CuSbS_2_/Ni | 1.095 | 22.58 | 87.39 | 21.62 |
| 13 | Al/FTO/ZnO/Na_2_AuGaBr_6_/V_2_O_5_/Ni | 1.193 | 26.52 | 86.48 | 27.38 |
| 14 | Al/FTO/ZnO/Na_2_AuGaBr_6_/MoTe_2_/Ni | 1.153 | 26.19 | 88.02 | 26.6 |
| 15 | Al/FTO/ZnO/Na_2_AuGaBr_6_/Sb_2_S_3_/Ni | 1.169 | 26.4 | 87.59 | 27.05 |
| 16 | Al/FTO/ZnO/Na_2_AuGaBr_6_/CuO/Ni | 1.105 | 23.82 | 87.83 | 23.13 |
| 17 | Al/FTO/WS_2_/Na_2_AuGaBr_6_/CFTS/Ni | 1.12 | 24.96 | 88.59 | 24.78 |
| 18 | Al/FTO/WS_2_/Na_2_AuGaBr_6_/CuI/Ni | 1.151 | 26.06 | 89.12 | 26.75 |
| 19 | Al/FTO/WS_2_/Na_2_AuGaBr_6_/NiO/Ni | 1.193 | 26.44 | 89.22 | 28.16 |
| 20 | Al/FTO/WS_2_/Na_2_AuGaBr_6_/CuSbS_2_/Ni | 1.098 | 23.09 | 87.85 | 22.28 |
| 21 | Al/FTO/WS_2_/Na_2_AuGaBr_6_/V_2_O_5_/Ni | 1.229 | 26.52 | 88.79 | 28.96 |
| 22 | Al/FTO/WS_2_/Na_2_AuGaBr_6_/MoTe_2_/Ni | 1.162 | 26.23 | 89.18 | 27.2 |
| 23 | Al/FTO/WS_2_/Na_2_AuGaBr_6_/Sb_2_S_3_/Ni | 1.183 | 26.42 | 89.23 | 27.91 |
| 24 | Al/FTO/WS_2_/Na_2_AuGaBr_6_/CuO/Ni | 1.108 | 24.17 | 88.33 | 23.67 |
| 25 | Al/FTO/C_60_/Na_2_AuGaBr_6_/CFTS/Ni | 1.117 | 24.25 | 85.38 | 23.14 |
| 26 | Al/FTO/C_60_/Na_2_AuGaBr_6_/CuI/Ni | 1.147 | 25.38 | 85.71 | 24.96 |
| 27 | Al/FTO/C_60_/Na_2_AuGaBr_6_/NiO/Ni | 1.183 | 25.78 | 85.4 | 26.05 |
| 28 | Al/FTO/C_60_/Na_2_AuGaBr_6_/CuSbS_2_/Ni | 1.095 | 22.35 | 84.65 | 20.73 |
| 29 | Al/FTO/C_60_/Na_2_AuGaBr_6_/V_2_O_5_/Ni | 1.206 | 25.85 | 84.73 | 26.44 |
| 30 | Al/FTO/C_60_/Na_2_AuGaBr_6_/MoTe_2_/Ni | 1.157 | 25.56 | 85.69 | 25.35 |
| 31 | Al/FTO/C_60_/Na_2_AuGaBr_6_/Sb_2_S_3_/Ni | 1.175 | 25.75 | 85.53 | 25.89 |
| 32 | Al/FTO/C_60_/Na_2_AuGaBr_6_/CuO/Ni | 1.105 | 23.44 | 85.11 | 22.07 |
| 33 | Al/FTO/IGZO/Na_2_AuGaBr_6_/CFTS/Ni | 1.03 | 24.72 | 83.43 | 21.26 |
| 34 | Al/FTO/IGZO/Na_2_AuGaBr_6_/CuI/Ni | 1.034 | 25.99 | 83.74 | 22.51 |
| 35 | Al/FTO/IGZO/Na_2_AuGaBr_6_/NiO/Ni | 1.035 | 26.43 | 83.85 | 22.95 |
| 36 | Al/FTO/IGZO/Na_2_AuGaBr_6_/CuSbS_2_/Ni | 1.024 | 22.57 | 82.82 | 19.16 |
| 37 | Al/FTO/IGZO/Na_2_AuGaBr_6_/V_2_O_5_/Ni | 1.035 | 26.51 | 83.87 | 23.03 |
| 38 | Al/FTO/IGZO/Na_2_AuGaBr_6_/MoTe_2_/Ni | 1.034 | 26.19 | 83.79 | 22.71 |
| 39 | Al/FTO/IGZO/Na_2_AuGaBr_6_/Sb_2_S_3_/Ni | 1.035 | 26.4 | 83.84 | 22.92 |
| 40 | Al/FTO/IGZO/Na_2_AuGaBr_6_/CuO/Ni | 1.027 | 23.81 | 83.2 | 20.37 |
| 41 | Al/FTO/In_2_S_3_/Na_2_AuGaBr_6_/CFTS/Ni | 1.108 | 24.82 | 86.38 | 23.77 |
| 42 | Al/FTO/In_2_S_3_/Na_2_AuGaBr_6_/CuI/Ni | 1.131 | 25.99 | 85.88 | 25.26 |
| 43 | Al/FTO/In_2_S_3_/Na_2_AuGaBr_6_/NiO/Ni | 1.151 | 26.41 | 84.93 | 25.83 |
| 44 | Al/FTO/In_2_S_3_/Na_2_AuGaBr_6_/CuSbS_2_/Ni | 1.09 | 22.81 | 86.09 | 21.42 |
| 45 | Al/FTO/In_2_S_3_/Na_2_AuGaBr_6_/V_2_O_5_/Ni | 1.158 | 26.48 | 84.49 | 25.94 |
| 46 | Al/FTO/In_2_S_3_/Na_2_AuGaBr_6_/MoTe_2_/Ni | 1.138 | 26.18 | 85.59 | 25.51 |
| 47 | Al/FTO/In_2_S_3_/Na_2_AuGaBr_6_/Sb_2_S_3_/Ni | 1.148 | 26.38 | 85.11 | 25.78 |
| 48 | Al/FTO/In_2_S_3_/Na_2_AuGaBr_6_/CuO/Ni | 1.099 | 23.97 | 86.32 | 22.76 |

**Table S2.** The work function values of left and right contact metals.

| Right Contact Metal | Ba | Ca | Mg | Al | Ag | Cr |
| --- | --- | --- | --- | --- | --- | --- |
| Work Function | 2.7 | 2.9 | 3.66 | 4.08 | 4.26 | 4.5 |
| Left Contact Metal | Co | Au | Ni | Pd | Pt | Se |
| Work Function | 5 | 5.3 | 5.5 | 5.6 | 5.7 | 5.9 |
